# Perinatal mAChR-mediated activation of cortical subplate neurons elicits network activity driving basket cell differentiation

**DOI:** 10.3389/fncel.2026.1799121

**Published:** 2026-05-14

**Authors:** Petra Wahle, Michelle Kaczmarski, Christian Riedel, Mohammad I. K. Hamad, Olga Arne, Erwan Dupont, Alexander Jack, Lisa-Marie Rennau, Andrea Räk, Heiko J. Luhmann, Ina Köhler, Silke Patz

**Affiliations:** 1 Developmental Neurobiology, Faculty of Biology and Biotechnology, Ruhr University, Bochum, Germany; 2 Institute of Physiology, University Medical Center, Johannes Gutenberg University, Mainz, Germany

**Keywords:** axogenesis, axon-carrying dendrites, dendritogenesis, fast-spiking and regular-spiking inhibitory neurons, pyramidal cell dendrites and spines

## Abstract

The activation of mAChR on subplate neurons has been shown to trigger oscillatory glutamatergic network activity in early postnatal neocortex. Here, we explored the long-term consequences of subplate activation on the differentiation of cortical neurons. Stimulating newborn somatosensory and visual cortex ex vivo and organotypic cortex cultures (OTCs) of the visual cortex with carbachol (CCH) led to an upregulation of *Bdnf* and *Zif-268*. In OTCs, the stimulation increased the amplitude and frequency of calcium events in the gray matter layers at DIV 3–5. At DIV 5, an acute stimulation rapidly activates p21ras; increases ERK phosphorylation; induces *Bdnf, c-Fos,* and *Zif-268* expression; and increases GluN1, GluN2A, GluN2B, PSD-95, GAD-65, and synaptophysin protein expression. The NMDA receptors were functional, as shown by enhanced ligand-induced dendritic injury. Repeated DIV 1–5 mAChR activation increased, at DIV 10, the expression of GluN2A, of an essential protein of the inhibitory presynapse, VGAT, and the basket-cell-specific presynaptic calcium sensor synaptotagmin-2. Furthermore, it increased the frequency of interneuronal calcium events at DIV 10–15, dendritic length of basket cells, and accelerated the local arborization of basket cell axons at DIV 15. The results suggest that temporally limited mAChR-mediated activation of subplate neurons evokes glutamatergic network activity, which, gradually over the following weeks, promotes cortical circuit development. Specifically, it enhances the expression of excitatory and inhibitory synaptic proteins and accelerates the morphological and functional maturation of basket cells, highlighting the cholinergic input to subplate neurons as a critical regulator of interneuron development.

## Introduction

Spontaneous neuronal activity represents a hallmark of the developing CNS. The rodent neocortex displays endogenously driven synchronous activity from before birth, with calcium events initially involving gap junctions and, subsequently, glutamatergic transmission. Around the second postnatal week, the maturing sensory systems ascending via the thalamus become the dominant drive (Garaschuk et al., 2000; Kilb and Luhmann, 2003; Dupont et al., 2006; Hanganu et al., 2009; Hanganu-Opatz, 2010; Wu et al., 2024). In the hippocampus, oscillations are evoked by the depolarizing action of GABA_A_ receptors (Leinekugel et al., 1997; Garaschuk et al., 1998; Kirmse et al., 2015), whereas visual cortex interneurons are inhibitory as early as postnatal day 3 and remain inhibitory during development (Murata and Colonnese, 2020). In the perinatal retina, events are driven by acetylcholine receptors (Feller et al., 1996), triggering synchronous activity and spindle bursts in visual cortex (Mooney et al., 1996; Hanganu et al., 2006). In the neocortex, oscillatory activity is triggered via muscarinic acetylcholine receptors (mAChR). The spread across the cortical layers initially involves gap junctions and, subsequently, ionotropic glutamate receptors (Garaschuk et al., 2000; Allène et al., 2008; Yang et al., 2009; Dupont et al., 2006; Kilb and Luhmann, 2003; Wu et al., 2024). This way, the activity of afferent systems shapes cortical maps (Antón-Bolaños et al., 2019).

Like many neuromodulators, ACh can also exert trophic roles, e.g., for hippocampal neurogenesis and cortical cytodifferentiation, which could well be mediated via neuronal depolarization (Hohmann et al., 1985; Hohmann et al., 1991; Johnston et al., 1995; Cooper-Kuhn et al., 2004; Mohapel et al., 2005; Bruel-Jungerman et al., 2011). ACh fibers innervate the cortex perinatally, ascending from the subplate to L6 and upper layers (Stichel and Singer, 1987), modulating activity via nicotinic and muscarinic receptors. Perinatal mAChR activation of subplate neurons with carbachol (CCH) elicits synchronous activity and up-states with bursts of action potentials and a glutamatergic transmission (Calderon et al., 2005; Peinado, 2000; Dupont et al., 2006; Sun and Luhmann, 2007; Hanganu et al., 2009; Luhmann et al., 2009). Ablating the subplate prevents the generation of synchronous activity and disrupts columnar development and GABA_A_Rα1 and KCC2 expression, resulting in an impaired maturation of cortical inhibition (Kanold and Shatz, 2006). In line, cholinergic deafferentation at birth disrupts expression of glutamate decarboxylase in inhibitory neurons and impairs pyramidal cell differentiation (Hohmann et al., 1985; Hohmann et al., 1991). Together, the early activity patterns are considered instructive for the maturation of connectivity, neurochemistry, and morphology, in particular of cortical GABAergic interneurons.

Numerous studies have employed activity deprivation to study the role of activity or have used lesions and deafferentation to test the role of transmitters. Lesions cause neuroinflammation, and for instance, perinatal exposure to the proinflammatory cytokine LIF impairs the maturation of cortical basket cells at the level of the presynapse (Engelhardt et al., 2018). An equally important approach is to activate the immature cortex in a biologically meaningful manner. For this, organotypic cortex cultures, an intact, long-living, spontaneously active *in vitro* model, are particularly suited. For instance, triggering network activity at DIV 7–10 with nanomolar kainate increases calcium events and promotes supragranular pyramidal cell dendritic development (Jack et al., 2019). Optogenetic stimulation at DIV 5–10 with a frequency matching the activity at the comparable developmental stage *in vivo* (Rochefort et al., 2009) promotes the maturation of pyramidal cell dendrites and increases the local arborization of basket cell axons (Gonda et al., 2023a; Gonda et al., 2023b). Moreover, axons of basket and non-basket interneurons arborize more rapidly with a DIV 5–10 pharmacological activation of GluN2D-containing NMDA receptors (Köhler et al., 2025b), which are enriched in interneurons (Booker and Wyllie, 2021). Here, we tested the hypothesis that perinatal activation of mAChR in subplate neurons will elicit depolarizing network activity in gray matter layers, which will accelerate the neurochemical and morphological maturation of cortical neurons.

## Materials and methods

### Ethic statement

All experiments were conducted in accordance with National and European (2010/63/EU) laws for the use of animals in research. Perinatal mouse cortex hemispheres ex vivo were explanted from animals born in the animal facility at the Johannes Gutenberg-Universität, Mainz, Germany. Perinatal rat (outbred pigmented Long-Evans of either sex) visual cortex was explanted from animals born in the animal facility at Ruhr-Universität, Bochum, Germany. According to the German Animal Welfare Act (“Tierschutzgesetz der Bundesrepublik Deutschland, TierSchG”, § 4), killing of vertebrates for sampling tissue or organs for scientific purposes does not require authorization. Therefore, no file numbers have been assigned to this project.

### Stimulation *ex vivo* and in organotypic cultures

The intact perinatal mouse neocortex was used *ex vivo*/*in vitro* for the bath-application of 8 pulses of CCH at 30 μM, washed every 20 min over a total of 3 h (Kilb and Luhmann, 2003; Dupont et al., 2006). We used this well-established model to test if this treatment could alter the expression of BDNF and immediate early genes.

Postnatal day 1 rat visual cortex was used to prepare roller-type organotypic slice cultures (OTCs). To examine interneuronal axonal maturation, the switch of rodent species allowed us to use reference data for interneuronal as well as for pyramidal cell morphology obtained during previous studies (Hamad et al., 2011; Jack et al., 2019; Gonda et al., 2020; Gonda et al., 2023a; Gonda et al., 2023b; Köhler et al., 2025a; Köhler et al., 2025b), in addition to validating the CCH effects in another species. Briefly, the visual cortex was explanted at P0-1 (P0 day of birth, ~5 pups per preparation) and chopped into 350 μm-thick slices with a McIlwain tissue chopper. Slices from every individual pup were allocated to the control and the CCH-treated group. Cultures were kept in medium containing 10% adult horse serum, 25% Hank’s balanced Salt Solution, 50% Eagle’s Basal Medium, 1 mM l-glutamine (all from Life Technologies, Karlsruhe, Germany), and 0.065% D-glucose (Merck, Darmstadt, Germany). The medium was changed every second day. To inhibit excessive glial growth, a mixture of anti-mitotic inhibitors (uridine, cytosine-ß-D-arabinofuranoside, and 5-fluorodeoxyuridine, all 10 μM final concentration, all from Sigma-Aldrich) was given at DIV 1 and removed after 24 h with a medium change.

To induce network activity in OTCs, CCH dissolved in water was supplemented from DIV 1–5 once daily in the medium for analysis at DIV 5. DIV 5 was selected because we knew that OTCs have glutamate-driven calcium events in gray matter neurons by that time (Hamad et al., 2011). For analysis at DIV 10, 15, and 20, cultures were switched at day 6 to normal medium with medium exchange twice weekly. Various concentrations of CCH, indicated in the figures, were tested for the experiments done to validate the OTC model. For acute stimulation, one pulse of CCH was given; culture age and pulse duration are mentioned in the figures. Toward the biochemical and the morphological readout, a 1 μM final concentration in the medium was used. For the dendritic injury assay, EGFP-transfected OTCs were exposed at DIV 5 for 10 min to 10 μM NMDA, followed by fixation and immunostaining. The mAChR antagonist atropine at 10 μM and the broad-spectrum NMDA receptor antagonist DL-2-amino-5-phosphonovaleric acid (APV) at 10 μM were incubated with the cultures for 5–10 min before the CCH stimulation. Control cultures were mock-stimulated with the same amount of water to guarantee equal mechanical influences and handling times outside the incubator.

### Protein blots

For the youngest age, OTCs were harvested a minimum of 6 h after the last CCH stimulation. OTCs were individually lysed in 20 μL RIPA-SDS buffer containing protease and phosphatase inhibitors as described, allowed to solubilize for 1 h at RT, and denatured for 20 min at 80 °C (Köhler et al., 2025b). The lysate of 1 OTC was run per lane. Proteins were separated on 10–12% SDS-PAGE gels and tank-blotted to 0.2 μm nitrocellulose (Schleicher and Schuell, or Amersham). Membranes were stained with PonceauS and cut into horizontal stripes spanning the kilodalton range for the target proteins as described, which permits detection of up to 10 proteins per lane/per culture (Engelhardt et al., 2018; Köhler et al., 2025b); β-actin or β-tubulin served as loading control and for normalization. For the phosphorylated epitopes, e.g., of glutamate receptors, 50% of each lysate, control, and CCH-stimulated, was loaded twice per gel, one half was developed for the total protein, and the other was developed with antibodies to the phosphorylated epitope. Band intensities were normalized to β-tubulin or β-actin of the same lane, and expressed relative to the control, which was set to 1. The normalized levels of the stimulated cultures were, gel-by-gel, expressed relative to the normalized values of the control cultures, the average of which was set to 1. The graphs and table report the mean with S. E. M. Statistical analysis was performed with the Mann–Whitney U-test with Sigma Plot 12 for Windows (SPSS Incorporated, Chicago, IL, United States).

For the pull-down of activated (GTP-bound) p21Ras, 10 OTCs per condition were pooled, lysed in proteinase-inhibitor-containing ice-cold lysis buffer, and centrifuged (13,000 *g*, 15 min, 4 °C). The supernatant was collected, and the protein concentration was determined with the Markwell assay. Active GTP-Ras was captured with a Raf1 kinase-derived, minimal Ras-binding domain peptide as described (Virdee et al., 1999; Heumann et al., 2000). After separating equal amounts of protein per lane, GTP-Ras and total Ras were detected by Western blotting. For MAP kinase/ERK1/2 blots, the tyrosine phosphorylation was detected first with chemoluminescence, followed by stripping and detection of the total ERK1/2 protein, and normalization to β-tubulin. Antibodies are mentioned in Supplementary Table 1.

### PCR

The CCH stimulation in P0/P1 intact mouse cortex hemispheres *ex vivo* was done as described (Dupont et al., 2006) at Mainz University (Luhmann lab) with 8 pulses of 30 μM CCH in aCSF over 3 h. The unstimulated hemisphere was maintained for 3 h in aCSF (containing (in mM) 124 NaCl, 26 NaHCO3, 5 KCl, 1.6 CaCl2, 1 MgCl2, 1.25 NaH2PO4, and 10 d-glucose, pH 7.4). Thereafter, the somatosensory and visual cortex were dissected from the hemispheres and frozen on dry ice. In addition, the areas were freshly dissected and immediately frozen in order to control for the effect of explantation. Tissue samples were blinded to condition and transferred to Ruhr University Bochum (Wahle lab). Here, tissue was lysed, and the mRNA was isolated with a Dynabead mRNA Direct Kit (Dynal, Hamburg, Germany) and used to synthesize cDNA libraries with Sensiscript reverse transcriptase (20 U/μL; Qiagen, Hilden, Germany) at 37 °C for 60 min. The somatosensory and visual cortex of every hemisphere delivered one lysate each, two to three reactions were run per lysate, normalized to *G6pdh* (*G6*), and averaged. The code was broken after all PCR reactions were completed.

For *in vitro* analysis, OTCs were harvested ~6 h after the last CCH stimulation. Always five OTCs per condition were pooled to generate one cDNA library, as reported and used for PCR analysis of all transcripts assessed. The single-band amplicons correspond to base pairs 451–1,072 for *M1*, 633–1,184 for *M2*, 591–1,380 for *M3*, 1,022–1,531 for *M4*, 1,199–1,220 for *M5* (Borges et al., 2001), 158–393 for *Bdnf*, 158–634 for *Nt3*, 309–605 for *Nt4*, 456–946 for *Ngf*, 1,422–1931 for *Trkb* kinase domain, 116–606 for *Trkb* extracellular ligand binding base, 1–448 for *c-Fos* and 740–1,039 for *Zif-268*; 713–1,085 for *Gad-65*, 713–1,159 for *Gad-67*, 21–381 for Neuropeptide Y (*Npy*), 137–363 for parvalbumin (*Parv*), and 2,112–2,272 for glucose-6-phosphate dehydrogenase (*G6pdh*, short: *G6*) as house-keeping product (Patz et al., 2003; Patz et al., 2004; Patz and Wahle, 2006). PCR conditions were kept within the exponential range determined for every product. PCR was normalized to *G6pdh* expression and expressed relative to the normalized values of the control condition, which were set to 1. The graphs show the mean with S. E. M. Statistical analysis was performed with the Mann–Whitney *U*-test (MWU-test).

### Expression plasmids and biolistic transfection

Cartridges were prepared by coating 7 mg gold particles (1 μm size; Bio-Rad, München, Germany) with 10 μg endotoxin-free plasmid encoding enhanced green fluorescent protein (EGFP) or mCherry as reporter under CMV promoter. Alternatively, the plasmid encoding pAAV-mDlx-GFP-Fishell-1 was used to enrich for interneurons and complete labeling of their axons. Cultures were transfected using a Helios Gene Gun (Bio-Rad, München, Germany) at 160–180 psi as described (Köhler et al., 2025a; Gonda et al., 2023a). Transfection was done at DIV 2–3 for analysis at DIV 5, at DIV 5–6 with analysis at DIV 10, or between DIV 5–10 with analysis at DIV 15 or DIV 20.

### Immunohistochemistry

For EGFP and mCherry staining, OTCs were fixed with 4% paraformaldehyde/0.1% glutardialdehyde in 0.1 M phosphate buffer, pH 7.4, warmed to 36 °C for 5 min, followed by 4% buffered paraformaldehyde for ~1 h. Fixation was done a minimum of 6 h after the last CCH stimulation. Immunostaining was done using a mouse-anti-EGFP and the ABC reaction as described (Köhler et al., 2025a; Gonda et al., 2023a). The DAB reaction product was intensified with 1% osmium tetroxide in phosphate buffer pH 7.4, followed by dehydration and coverslipping. For GABA and parvalbumin staining, OTCs were fixed with 4% phosphate-buffered paraformaldehyde with 0.3% glutardialdehyde for 15 min and postfixed for another 60–90 min with 4% paraformaldehyde. After washing, permeabilization with 1% Triton X-100 in PBS for 60 min, blocking with bovine serum albumin and normal goat serum in TBS, specific antibodies were incubated overnight at 4 °C, followed by biotin-conjugated secondaries, ABC reagent, and intensification of the DAB reaction product. NADPH staining was done to detect the subset of nitric oxide synthase-positive interneurons. OTC were incubated in the reaction solution [10 mg NADPH, 2.5 mg NBT, and 0.3% Triton X-100 per 10 mL 100 mM Tris–HCl buffer pH 7.4] at 37 °C for a minimum of 30 min until the somata were completely labeled, rinsed, and coverslipped. Phospho-ERK1/2 and NeuN were double-labeled with immunofluorescence followed by confocal imaging. Antibodies and reagents are listed in Supplementary Table 1.

### Neuron reconstruction and statistical analysis

GABA-stained and nitric oxide synthase (NADPH) positive somata were reconstructed with fineliner drawings using a Camera lucida at 1,000× magnification in 2–3 non-overlapping perpendicular stripes from layer (L) 1 down to L6. This way, 100–150 somata were sampled per OTC by observers blinded to condition. The somatic area [in μm^2^] was determined with ImageJ; the average per OTC was plotted. The density of parvalbumin-positive somata was determined at DIV 15 by plotting labeled somata in pie-slice sectors in the middle of the OTC from the pial surface to the white matter. On average, 1.6 mm^2^ were assessed per OTC; density was expressed as cells/mm^2^. EGFP-immunostained cells were reconstructed with the Neurolucida^®^ system (MicroBrightField, Williston, United States) at 1,000× magnification by trained observers blinded to conditions. Reconstructions were cross-checked by a different observer who was also blinded to conditions.

Established criteria were employed to classify pyramidal cells and interneurons by dendritic and axonal patterns (Karube et al., 2004; Hamad et al., 2011; Gonda et al., 2023a; Gonda et al., 2023b; Köhler et al., 2025b). Neurons were classified as pyramidal cells of L2/3 with an apical dendrite that reaches L1 and as pyramidal cells of L5/6 with an apical dendrite that ends in the middle layers. For pyramidal cells, apical dendritic length and segment number, mean length and segment number of all basal dendrites (≥30 μm length included), and the number of primary dendrites were assessed. For pyramidal cells, spine density (protrusions per 100 μm dendritic length) was quantified in L2/3 and L5/6 pyramidal cells at DIV 10, and in L2/3 pyramidal cells also at DIV 20 on several apical oblique branches and on basal dendrites starting distally to the first branch point (on average 250 μm length from 3 basal dendritic segments per cell); thick shafts and the tuft were not sampled. Additionally, the proportions of mushroom spines, thin spines, and filopodia were determined on secondary apical branches of L2/3 pyramidal cells.

Slice cultures prepared from perinatal cortex offer the advantage that interneurons differentiate entirely *in vitro*, and sparse transfection permits addressing the complete 3D axonal pattern. The interneuron types become distinguishable around 10 days after birth *in vivo* and similarly in organotypic cultures. We employed the morphological criteria demonstrated in recent work (Gonda et al., 2023b; Köhler et al., 2025b), and axonal ramification, in addition to synaptic target selectivity, is a decisive feature for classifying interneuron types (Ascoli et al., 2008; Staiger et al., 2015). Neurochemical markers were not employed, first, because at DIV 10, not all cells will express the adult markers (e.g., parvalbumin) at detectable levels, and second, the axonal analyses require completely stained cells, achievable only with optimal EGFP staining. We focused on local and horizontally projecting basket cells and vertically projecting translaminar non-basket cells with bitufted and arcade-shaped axons. Basket cells are fast-spiking interneurons targeting somata and proximal dendrites, whereas non-basket cells are primarily dendrite-targeting with an adapting firing pattern. Basket cells typically feature thick main axons and delicate collaterals dotted with irregular-sized boutons and terminal elements contacting somata as extensively demonstrated (Gonda et al., 2023b). As mentioned in the previous work, Martinotti cells were excluded due to their highly variable collateral extent in L1, bipolar cells due to their rather diffuse axon plexus, and neurogliaform neurons. Axons were analyzed with linear axograms, counting line crossings of axonal collaterals with 100 μm bins starting at the origin of the axon, as well as Sholl analyses in bins of 50 μm radius. Furthermore, the axonal growth response was analyzed separately for axons emerging from basket cell somata or from a dendrite. These dendrites have been termed the “axon-carrying dendrite, AcD”, and “AcD cell” (Thome et al., 2014; Wahle et al., 2022; Gonda et al., 2023b; Köhler et al., 2025b).

For interneuronal dendrites (≥30 μm length), the mean dendritic length, mean number of dendritic segments, and number of primary dendrites were determined. The latter were variable in number and not regulated by treatment, neither for pyramidal cells nor for interneurons. Furthermore, the density of dendritic protrusions was assessed for randomly selected basket and non-basket cells. To determine the presynaptic bouton size at DIV 15, completely EGFP-stained baskets in a solitary position were selected to minimize interference from bypassing axon collaterals of other cells. Photomicrographs of the axonal plexus and parvalbumin terminal fields were taken at 1,000× magnification with a CCD camera mounted to a Zeiss Axiophot microscope equipped with differential interference optics at random positions and focal depths within the dendritic field (20–30 ROI/OTC). The area of the boutons was determined with MacBiophotonics (ImageJ) by experimenters blinded to condition.

Statistical analysis was performed with Sigma Plot 12 for Windows (SPSS Incorporated, Chicago, IL, United States). Non-parametric Mann–Whitney *U*-tests or *t*-tests, as mentioned in the figure legends, were employed to assess statistically significant differences between the treatment group and control. Data were plotted either as bar graphs (showing the mean ± S. E. M.) or as box plots (box with median, 25%/75% quartiles, whiskers at 10%/90%).

### Calcium imaging

For 2-photon recordings of OTCs at DIV 3–5, we used Oregon Green 488 BAPTA-1 a.m. because transfection and expression of a genetically encoded calcium indicator could not be done reliably with such immature cells. We loaded 10 μM OGB-1 a.m. dissolved in 20% pluronic acid/DMSO (Molecular Probes, Eugene, OR, United States) diluted in 200 μL self-conditioned culture medium for 1 h at 36 °C (Hamad et al., 2011; Hamad et al., 2014). Excess dye was washed out with oxygenated ACSF (in mM: 125 NaCl, 2.5 KCl, 2 CaCl2, 1 MgSO4, 25 NaHCO3, 1.25 NaH2PO4, 10 d-glucose, pH 7.4). OTCs were incubated for another hour for recovery, then transferred to the recording chamber mounted on a fixed stage of an upright microscope and continuously perfused with oxygenated ACSF (3–5 mL/min at 32 ± 2 °C). Fluorometric recordings of spontaneously occurring calcium events of many infragranular neurons of the cortical gray matter were made using a custom-built two-photon laser-scanning microscope equipped with a Ti:sapphire laser system (Spectra-Physics, Mountain View, CA, United States) for mode-locked laser light (pulse width <100 femtoseconds; repetition rate, 80 MHz), a laser-scanning system attached to a movable objective system on an upright microscope (Sutter Instruments, Lambrecht/Pfalz, Germany), and a water-immersion objective (20x, 0.8 NA, Zeiss, Oberkochen, Germany). Scattered infrared light was blocked by means of a 680 nm barrier filter (Chroma Technologies, Rockingham, VT, United States) mounted in front of the detector. To monitor calcium events, the excitation wavelength was changed to 820 nm, the laser power was reduced (<80 mW), and emission was collected through a 535/550 nm band pass filter. Images were acquired at 4 frames/s and analyzed (MacBiophotonics ImageJ software; ScanImage 3.0). In the OGB-1 a.m. recording channel, individual cell somata were selected as a region of interest (ROI), and the mean intensities in the ROIs in each frame were determined. Raw data delivered in the form of a linear 16-bit intensity scale were first plotted as fluorescence intensity versus time. The background fluorescence measured near an ROI was then subtracted from these raw data. The F^0^ (resting fluorescence) image was generated by averaging the fluorescence intensities of 10–50 frames from the image stack in a time window where there was no activity (by eye inspection). Subsequently, data were normalized to the mean fluorescence intensities (summary formula Δ*F*/*F*^0^ = *F* –*F*^0^/*F*^0^), allowing the comparison of data across experiments using Mann–Whitney *U*-tests.

For the confocal calcium imaging at DIV 10–15, we initially used OGB-1 with transfection of mCherry to identify and record neuronal transfectants. We also co-transfected mCherry with a genetically encoded calcium sensor, the pAAV-mDlx-GCaMP6f-Fishell-2 construct, to enrich for interneurons. Activity of selected neurons was recorded with a Leica TCS SP5 confocal microscope (Leica, Mannheim, Germany) with a 10x objective at 1400 Hz and 2.7 frames/s as described (Köhler et al., 2025a). Spontaneously occurring calcium events were recorded for 10–15 min in cultures from >6 independent preparations. The maximal calcium event amplitude was determined, and the duration of events (defined as rise and decline to baseline fluorescence) was measured at half-maximal width. Raw data were delivered in the form of a linear 16-bit intensity scale versus time and analyzed using MacBiophotonics ImageJ software. Normalizing Δ*F*/*F*_o_ was done to eventually pool the recordings with OGB-1 and GCaMP6f and to plot the data with the average of the controls set to 1. Z-stacks of the mCherry-fluorescent cells were taken after recording. Then, cultures were fixed, immunoperoxidase-stained for mCherry or for GCaMP6 (using the EGFP antibody), and the recorded neurons were identified in the maximum projection image, classified by the immunostaining as pyramidal cell or interneuron, followed by reconstruction of dendrites and, partially, of the axonal plexus.

## Results

### Acute mAChR activation of *ex vivo* subplate/L6b neurons promotes expression of immediate early genes in gray matter neurons

**Figure 1:**
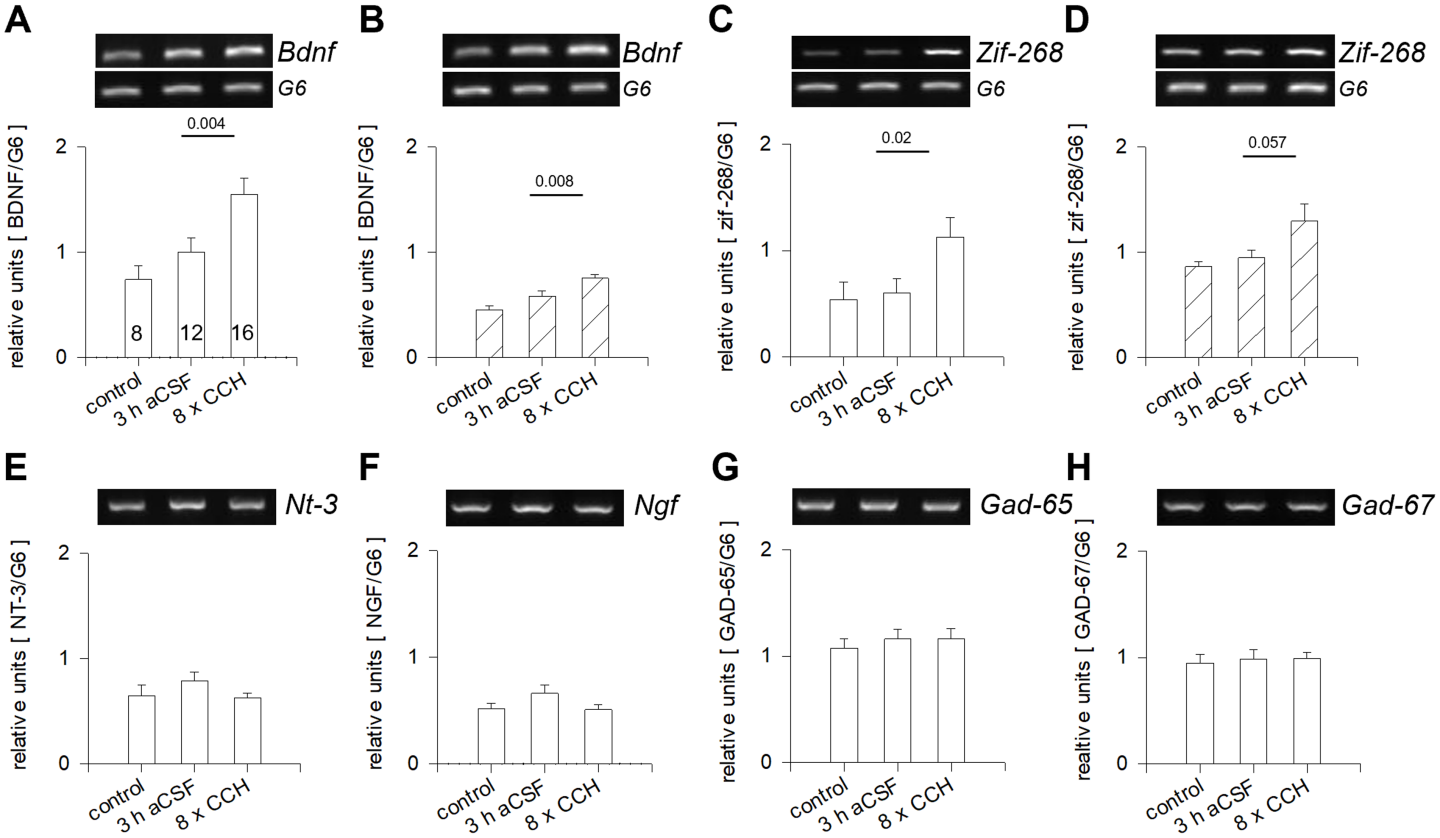
*Ex vivo* CCH stimulation of P0-P1 mouse cortex evokes Bdnf and Zif-268 transcription. RT-PCR analysis of CCH-stimulated somatosensory and occipital cortex (hatched bars) in comparison to freshly dissected cortex (bars labeled control) or cortex from hemispheres kept in aCSF for 3 h without stimulation. **(A,B)** Bdnf; **(C,D)** Zif-268; **(E)** *Nt-3*, **(F)** *Ngf*; **(G)** *Gad-65*; **(H)** *Gad-67*. The number of hemispheres per condition is given in **(A)**. Mean ± S. E. M.; MWU-test comparing aCSF versus aCSF+CCH; *p*-values are indicated.

P0–P1 intact mouse cortex hemispheres ex vivo respond to CCH with spindle oscillations and an increase of glutamatergic network activity (Dupont et al., 2006). We repeated this protocol to subject somatosensory and visual cortex to PCR analysis (Figure 1). The 3 h aCSF control condition subtly increased the mRNA expression of *Bdnf*, but not *Zif-268*, in comparison to freshly dissected hemispheres, presumably due to lesioning (Figures 1A,B). CCH stimulation with 8 pulses over 3 h increased *Bdnf* and *Zif-268* mRNA expression in both cortices in comparison to the 3 h aCSF condition (Figures 1A–D). Induction of *Zif-268* is mediated by m1 activation (Ebihara and Saffen, 1997). The higher baseline and stronger upregulation of Bdnf mRNA in the somatosensory cortex compared to the visual cortex are likely due to the advanced maturity of the somatosensory cortex at birth. In the newborn cortex, *Nt-3*, *Ngf*, *Gad-65/67* mRNA (Figures 1E–H) were not changed. The results suggested that acute stimulation with CCH quickly upregulates activity-dependent *Bdnf* and *Zif-268* transcription, both known to promote cortical maturation. Next, OTCs of the visual cortex were validated as a model for assessing the long-term effects of a perinatal mAChR activation of the subplate.

Subplate neurons comprise GABAergic local axon cells and inverted excitatory pyramidal cells, which project in a columnar manner into the gray matter (Friauf et al., 1990; Naegele et al., 1991), serving a role as instructive ‘helper’ neurons and integrators of spontaneous and sensory-driven activity (Molnár et al., 2020). In OTCs, ongoing cellular migration and the flattening of the roller-type slices cause an intermingling of subplate/L6b and L6 proper such that the deepest pyramidal cells can no longer be safely addressed as subplate cells by morphology. An iconic subplate cell type in cat, pig, and primate is the axonal loop cell with long-range projections ascending to L1 (Wahle and Meyer, 1987; Uylings and Delalle, 1997; Ernst et al., 2018). Axonal loop cells have recently been shown to be immunonegative for GAD-65/67 and thus are excitatory and well-suited to elicit network activity over larger distances. Axonal loop cells were present among the EGFP transfectants in OTCs residing adjacent to the remains of the white matter (Supplementary Figure 1A). The neuron had already had mild symptoms of dendritic beading suggestive of degeneration. The axon branched into numerous collaterals extending in the white matter and ascending into the upper layer, passing in close apposition to dendrites and axons of pyramidal and non-pyramidal neurons of L2-6, suggestive of synaptic or humoral transmission (Supplementary Figure 1B).

### Effective concentration and kinetics of CCH stimulation of OTCs

**Figure 2:**
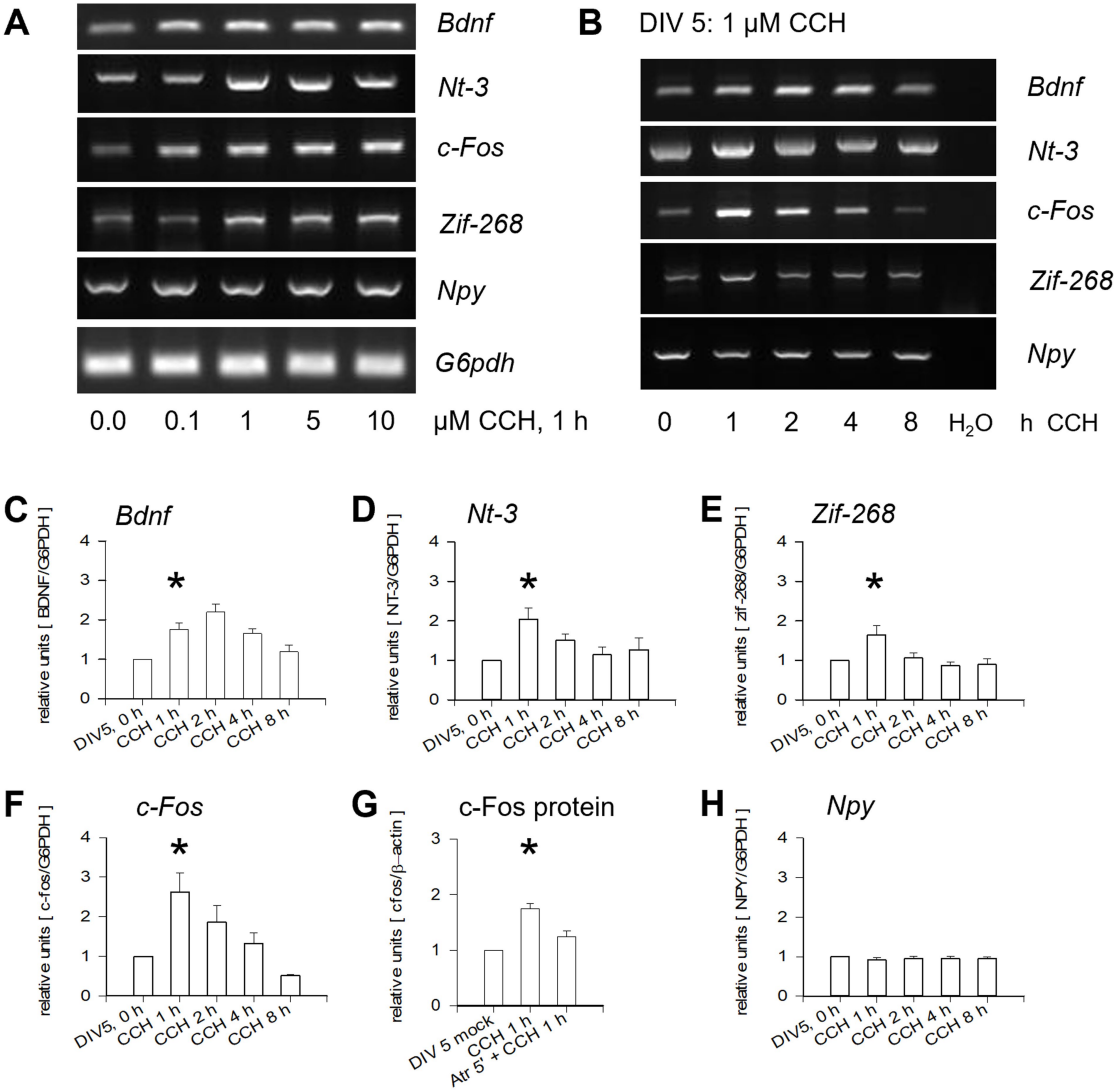
Activation of mAChR increases mRNA expression in DIV 5 OTCs. **(A)** Dose-dependent upregulation of *Bdnf*, *Nt*-3, *c-Fos*, *Zif*-268, but not *Npy*. Already 1 μM CCH elicited expression levels 1.5–2 fold over control, with no further increase by 5 or 10 μM CCH. **(B)** Kinetics revealed transient increases in mRNA expression after one pulse of 1 μM CCH for the times (in hours) indicated; lane labeled H_2_O represents reactions without template cDNA. **(C–F,H)** Quantification of **(C)** *Bdnf*; **(D)** *Nt*-3; **(E)** *Zif*-268; **(F)** *c-Fos* mRNA. **(G)** C-Fos protein was also increased, and this was sensitive to atropine (Atr). **(H)** *Npy* remained constant. Three lysates each of 5 OTCs per time point, 3 reactions per lysate, values normalized to G6PDH and averaged. Mean ± S. E. M., DIV 5 mock-stimulated control was set to 1; *t*-test for 1 h time point versus mock-stimulated control with *p < 0.05.

During preparation, OTCs become deprived of the cholinergic afferents. This makes OTCs an ideal system to probe the effects of CCH on mAChR signaling, particularly the depolarizing m1 receptors (Calderon et al., 2005; Dupont et al., 2006; Hanganu et al., 2009). First, dose-dependency and activation kinetics of selected mRNAs in OTCs were assessed; all known to be downstream of depolarizing transmission and calcium influx. The mRNA of *Bdnf*, *Nt-3*, *c-Fos*, and *Zif-268* were increased after 1 h exposure to CCH, whereas *Npy* mRNA remained constant (Figure 2A). Notably, concentrations as low as 1 μM already induced maximal effects (Figure 2A and Supplementary Figures 2A–E) in line with earlier reports (Shinoe et al., 2005). This dose was selected, since higher concentrations could downregulate m1 expression (Wang et al., 1994). A single pulse of 1 μM CCH increased *Bdnf*, *Nt-3*, *and c-Fos* mRNA expression, which lasted for hours (Figures 2B–F). Moreover, c-Fos protein was increased, and this was sensitive to atropine (Figure 2G). The increase of *Zif-268* was limited to the first hour (Figure 2E). No change was seen for *Npy* expression (Figures 2B,H). The results indicate that early mAChR activation of subplate neurons evokes a sustained activation of neurotrophic signaling and transcription factors reminiscent of effects reported for early environmental enrichment.

### Effects of repeated mAChR activation of subplate/L6b neurons

Next, a DIV 1–5 CCH stimulation (1 pulse daily for 5 days) was tested. OTCs were harvested at DIV 5, a minimum of 3 h after the last pulse of CCH. Other OTCs from the same batches were maintained in normal medium without further stimulation until harvest at DIV 10 and DIV 20. At DIV 5, only *Bdnf* and *c-Fos* mRNA were increased (Supplementary Figures 3A,G). At DIV 10 and 20, transcript levels in stimulated OTC were indistinguishable from controls (Supplementary Figures 3A–L). As expected, several transcripts became ontogenetically upregulated, e.g., *Gad-65/67* and *Parvalbumin* (Supplementary Figures 3B,C,G,I–K), whereas *TrkB* remained constant (Supplementary Figures 3E,F), and *Nt-3* and *Npy* declined later in development. The absence of NPY induction is notable, as hyperactivity is known to rapidly trigger NPY produced as an endogenous anticonvulsant (Cattaneo et al., 2021). This indicated that early mAChR activation of subplate neurons evokes physiological, not pathophysiological, network activity.

### Acute mAChR activation of subplate/L6b neurons evokes ERK phosphorylation

**Figure 3:**
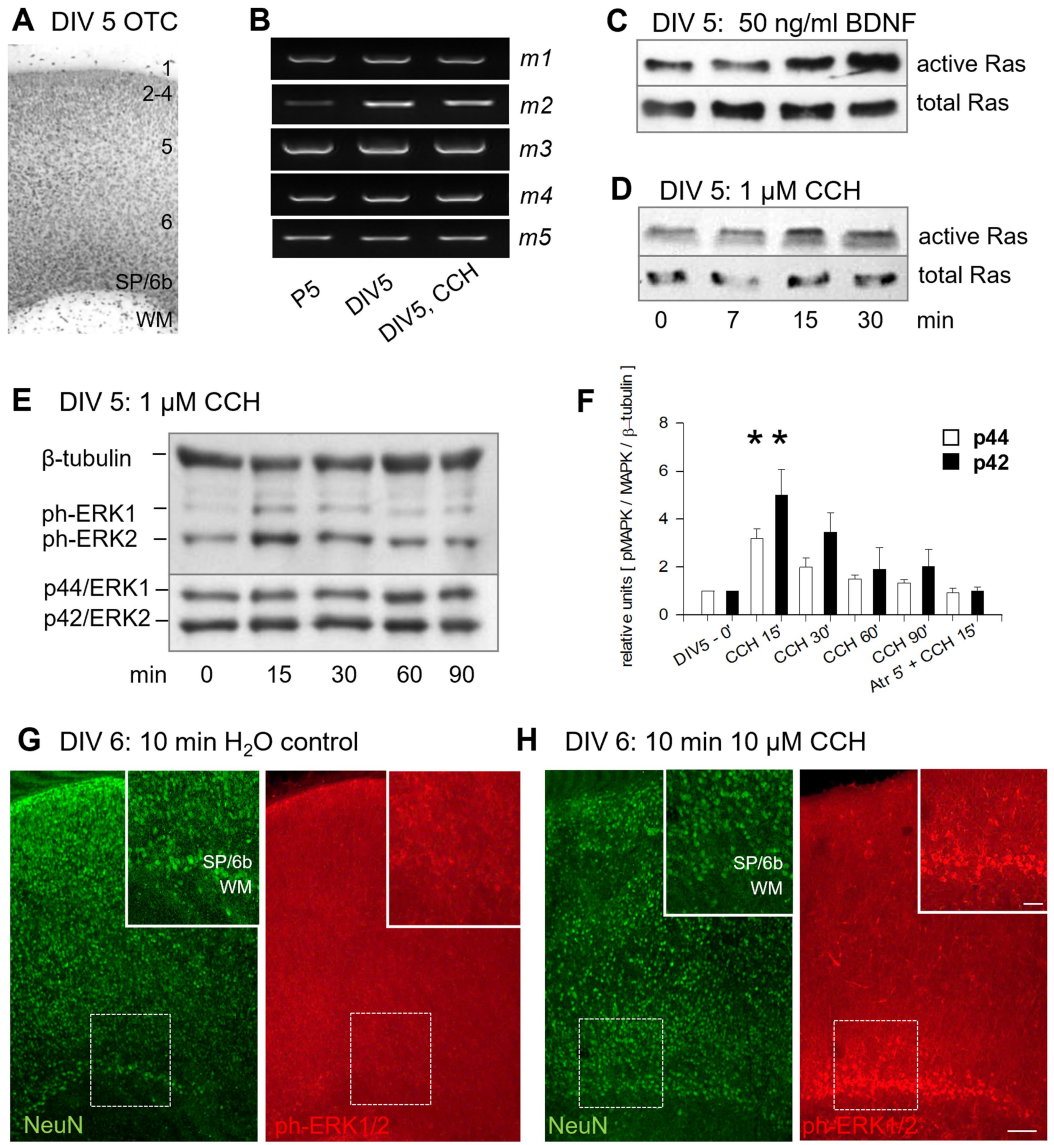
Activation of mAChR of subplate/L6b neurons triggers signaling cascades in young OTCs. **(A)** Thionin-stained OTC. **(B)** M1-5 expression in rat visual cortex at P5 and DIV 5 OTCs. The mRNAs were not regulated by CCH. **(C)** At DIV 5, a pulse of BDNF activates p21Ras. **(D)** Similarly, a pulse of CCH activates p21Ras (*n* = 3 pull downs with 10 OTCs each; mean ± SD: 1.49 ± 0.26 over control). **(E)** CCH at 1 μM activated MAP kinase/ERK1/2 phosphorylation. **(F)** ERK1/2 phosphorylation peaked at 15 min, significant for each of the isoforms (*t*-test *p < 0.05 versus each control DIV 5–0′). CCH failed in the presence of atropine (Atr). Three lysates of 5 OTCs per time point, each run twice for the kinetic; 6 individual OTC lysates for the atropine experiment; shown are the mean ± S. E. M. Expression plotted as relative units normalized to mock-stimulated controls per blot; the average of the controls was set to 1. **(G)** Phospho-ERK1/2 immunofluorescence was weak in mock-stimulated OTCs; co-labeled with NeuN. **(H)** Phospho-ERK1/2 immunofluorescence was strong in subplate/L6b neurons 10 min after CCH stimulation. Insets show the boxed areas at higher magnification. Scale bars: 100 μm for the overviews, 50 μm for the insets.

Thionin-staining of DIV 5 OTCs revealed an organotypic layering with cell-sparse L1 and former white matter, suggesting the presence of subplate/L6b (Figure 3A), as supported by the presence of axonal loop cells (Supplementary Figure 1). Induction of early network activity requires m1/m5-containing mAChR (Hanganu et al., 2009), known to become expressed also in cultured neurons (Eva et al., 1990). Indeed, *m1-m5* were expressed at DIV 5 at levels comparable to age-matched visual cortex *in vivo*; only *m2* was slightly higher. None was altered by the DIV 1–5 CCH stimulation (Figure 3B).

A single 1 μM CCH pulse at DIV 5 efficiently activated signaling cascades: pull-down assays showed increased p21Ras activation at 15 and 30 min, comparable to BDNF stimulation (Figures 3C,D). Within minutes, a single pulse of CCH activated p42/44 ERK1/2 phosphorylation, which then declined over 90 min (Figures 3E,F). This was sensitive to atropine, indicating the involvement of mAChR (Figure 3F, last pair of bars). The m1 receptor drives MAP kinase activation in cortical stem cells explanted at E13, and m1 signaling evoked by 100 μM CCH contributes to sustained MAP kinase activation in a Src and NMDAR-dependent manner in pyramidal cells in vivo and *in vitro* (Rosenblum et al., 2000; Ma et al., 2000; Hamilton and Nathanson, 2001; Berkeley et al., 2001). Moreover, calcium influx via the NMDA receptors induces expression of immediate early genes via a MAP kinase-dependent mechanism (Xia et al., 1996). Muscarinic binding sites are present in the rat subplate on the day of birth (Schlumpf et al., 1991). An 8–10 min exposure to CCH at 10 μM final concentration, applied to surpass immunofluorescence detection thresholds, elicited strong phospho-ERK labeling in NeuN-positive subplate/L6b neurons (Figures 3G,H). Some labeled cells were already seen in the gray matter layers, presumably due to glutamatergic action potentials rapidly transmitted by ascending projections. In other words, subplate/L6b neurons were the first to strongly respond to one pulse of low-dose CCH and trigger a signaling cascade in the gray matter.

### mAChR activation of subplate/L6b neurons promotes activity in gray matter neurons

Recordings in intact mouse hemispheres ex vivo have extensively characterized the activity elicited by mAChR activation of the subplate. It reliably evokes action potentials in subplate neurons, leading to enhanced activity in gray matter neurons. The activity is synchronized in a columnar fashion via gap junctions, is spreading over the cortex, and, toward the end of the first postnatal week, is mediated by glutamate receptors without involvement of GABA receptors (Dupont et al., 2006; Kilb and Luhmann, 2003). Other studies have confirmed the role of NMDA and AMPA-type glutamate receptors for eliciting and spreading of early cortical network activity (Garaschuk et al., 2000; Allène et al., 2008; Yang et al., 2009).

**Figure 4:**
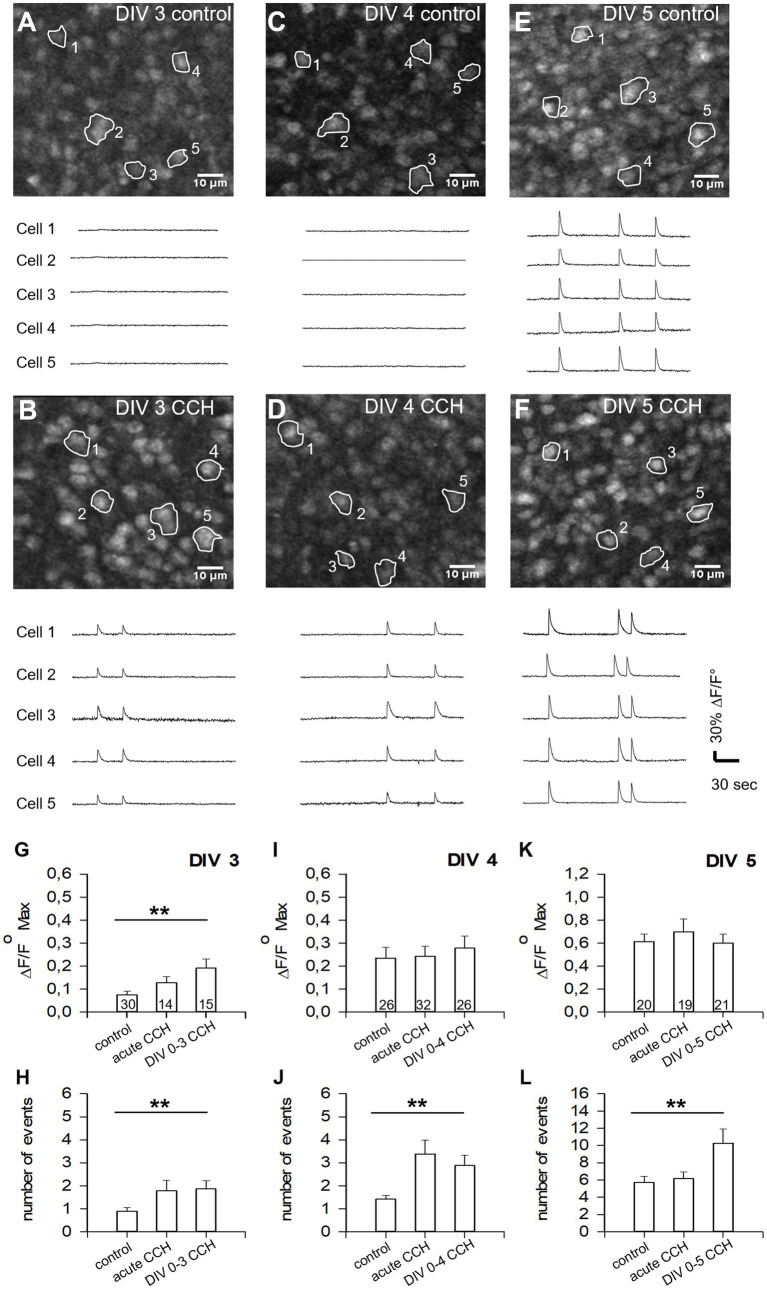
Activation of mAChR of subplate/L6b neurons evokes activity in young OTCs. **(A–F)** Calcium imaging in OTCs aged DIV 3, 4, and 5 revealed that CCH stimulation from the day of preparation **(B,D,F)** elicits calcium events in gray matter neurons and triggers synchronous activity at times when control OTCs are still largely silent. The five arbitrarily selected somata in the imaging frame delivered the calcium traces shown below each frame. **(G–L)** Quantification of amplitude **(G,I,K)** and frequency **(H,J,L)** of calcium events in mock-stimulated cultures (control), after 1 pulse of CCH (acute), and in OTCs stimulated from DIV 0 with 1 μM CCH once daily. Shown are mean ± S. E. M.; the number of ROI (5 cells/ROI, averaged) analyzed is indicated in the bars. MWU-test control versus the repeated stimulation; **p < 0.01. OTCs were from 4 preparations, 21 OTCs used at DIV 3, 21 OTCs at DIV 4, and 14 OTCs at DIV 5.

As proof-of-concept, we demonstrate that perinatal mAChR activation promotes the electrical activity in young neurons of L4-6 (Figure 4). OTCs were stimulated with one pulse of 1 μM CCH daily starting for this experiment from DIV 0–3, DIV 0–4, and DIV 0–5 with recording at DIV 3, DIV 4, and DIV 5 about 6 h after the last pulse. Compared to mock-stimulated controls (Figures 4A,C,E), the CCH-stimulated OTCs (Figures 4B,D,F) displayed higher levels of calcium events in individual cells already at DIV 3 (Figures 4G,H). At DIV 4 and DIV 5, amplitudes were not altered, but the frequency of calcium events was higher than in control (Figures 4I–L). In addition, an acute (1 pulse) stimulation with 1 μM CCH was done at DIV 3, 4, and 5 with OTCs from the same batches (bars labeled ‘acute CCH’ in Figures 4G–L). The acute stimulation also increased amplitude and frequency at DIV 3 and frequency at DIV 4. Together, the repeated CCH stimulation until DIV 5 resulted in a higher number of calcium events at DIV 5. Earlier work in OTCs has shown that, at DIV 5, AMPA receptor antagonists are able to block spontaneous calcium events in gray matter neurons (Hamad et al., 2011) in line with previous reports (Garaschuk et al., 2000; Allène et al., 2008; Yang et al., 2009). The result confirmed that calcium events in gray matter neurons can be increased in number with 1 μM CCH via an enhancement of glutamatergic signaling from excitatory subplate neurons projecting into gray matter layers.

### Acute activation of mAChR of subplate/L6b neurons increases selected proteins in an age-dependent manner in gray matter neurons

**Figure 5:**
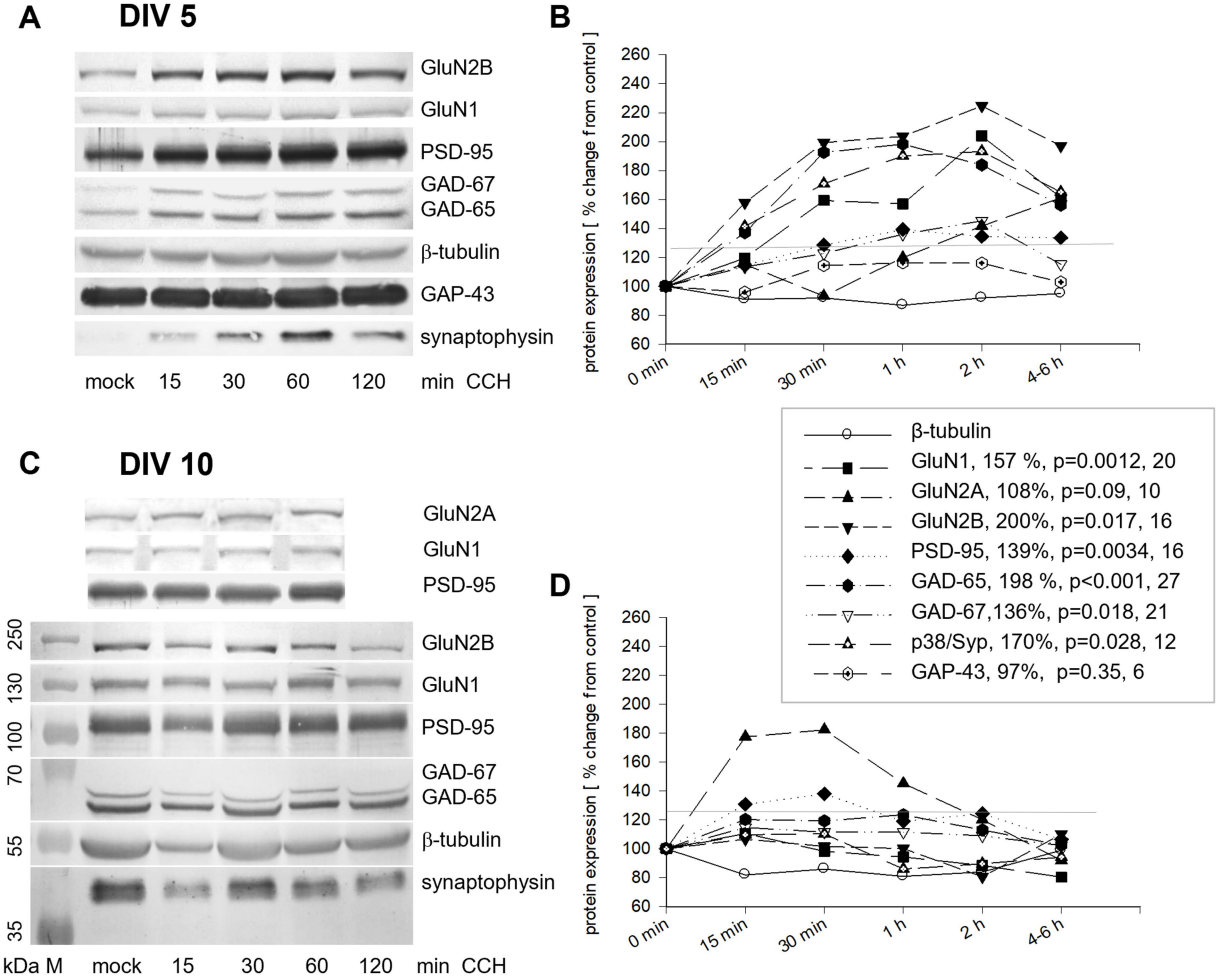
Acute activation of mAChR elicits short-term changes of protein expression in an age-dependent manner. **(A,B)** Acute stimulation with one 1 μM pulse of CCH at DIV 5. **(A)** Representative blot from one gel showing the increase of GluN2B, GluN1, PSD-95, GAD-65, GAD-67, and p38/synaptophysin, but not GAP-43 and β-tubulin. **(B)** Quantification of the proteins at the time points indicated. The average of the controls was set to 100% (abscissa); the ordinate shows relative changes in expression. One culture per lane, normalization to β-tubulin. For the curves in **(B)**, changes were statistically tested at the time point 1 h after the pulse with the MWU-test versus control. The per cent increase, the *p*-value, and the number of cultures assessed at the 1 h time point are given in the graphical legend (legend accounts for **B,D**) inserted between the graphs. **(C,D)** Effects of an acute stimulation with one 1 μM pulse of CCH at DIV 10. **(C)** Representative blots show the increase of GluN2A. M, marker lane. Note that either GluN2A or GluN2B can be detected per blot because both proteins run at ~180 kDa. **(D)** At DIV 10, only the GluN2A protein increased at 15 and 30 min to nearly 180%, and declined within 2 h. The 125% threshold is indicated by gray lines in **(B,D)**.

Many proteins for neurochemical maturation and synaptic transmission are developmentally upregulated in the early postnatal cortex, often in dependence on glutamatergic signaling. OTCs were stimulated at DIV 5 with one pulse of 1 μM CCH, harvested at 15, 30, 60, and 240/360 min. Protein blots revealed a substantial increase of GluN2B, GluN1, p38/synaptophysin, and GAD-65, which lasted for hours before declining. PSD-95 and GAD-67 showed smaller increases, whereas GluN2A exhibited only a modest increase at the latest time point. To distinguish true effects from noise (biological or experimental variability), changes were considered meaningful when exceeding 125% of baseline (threshold line in Figures 5B,D) for two time points. GAP-43 detected in the same lysates remained constant, as was β-tubulin (Figures 5A,B). Strikingly, stimulation with one pulse of 1 μM CCH at DIV 10 transiently increased only GluN2A (Figures 5C,D). The results suggested that a single pulse of CCH at DIV 5 and at DIV 10 can elicit a rapid, presumably translational activation of select proteins and that the consequence of mAChR activation changes with age.

### Activation of mAChR of subplate/L6b neurons evokes longer-lasting increases of proteins in the gray matter

**Figure 6:**
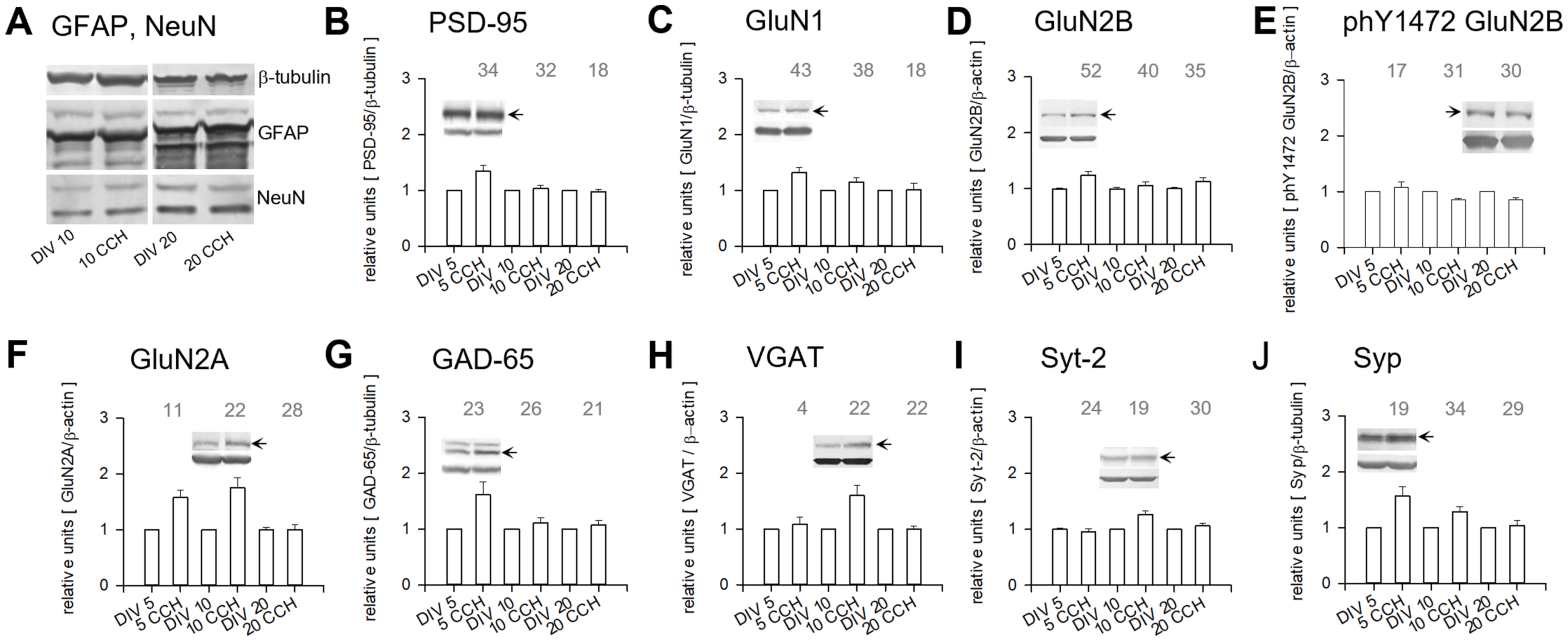
DIV 1–5 mAChR activation evokes longer-lasting changes of protein expression. DIV 1–5 CCH-stimulated OTCs harvested 6 h after the last pulse at DIV 5, at DIV 10, and at DIV 20, paired with mock-stimulated controls from the same batch. Lysates of control and treated cultures (1 OTC per lane) were run on the same gel for quantification. The number of stimulated OTCs analyzed for every protein is given above the bars (the total number of OTCs, of course, is twice that number). **(A)** Astrocytic GFAP and neuronal NeuN were unchanged. Upregulated at DIV 5 were: **(B)** PSD-95; **(C)** GluN1; **(D)** GluN2B; **(G)** GAD-65, but not GAD-67 (upper band). **(E)** Phosphorylation at Y1472 of GluN2B was reduced at DIV 10 and DIV 20. **(F)** GluN2A and **(J)** p38/Syp were upregulated at DIV 5 and DIV 10. **(H)** VGAT-1 and **(I)** Syt-2 were upregulated at DIV 10. Graphs show mean ± S. E. M, representative bands of the target protein (arrow) were cropped from the blot scans used for densitometry and arranged with the house-keeping protein band (always the lower bands) from the same lane above the selected pair of bars.

The next question was whether a repeated DIV 1–5 CCH stimulation could elicit similar changes of protein expression, and—if yes, were they long-lasting? DIV 5 OTCs were harvested 6 h after the last CCH pulse. Other DIV 1–5 CCH-stimulated OTCs were continued in normal medium without further stimulation until DIV 10 and DIV 20. Mock-stimulated control OTCs from the same batch were kept for the same periods of time (Figure 6). The DIV 1–5 stimulation did not obviously influence the expression of GFAP and NeuN at DIV 10 and 20 (Figure 6A), suggesting no changes in neuronal survival or astrocytic proliferation. Interestingly, the repeated stimulation increased the same set of proteins as the acute stimulation, including PSD-95, GluN1, GluN2A, GluN2B, GAD-65, and p38/synaptophysin (Figures 6B–G), in addition to VGlut-1 and synaptotagmin-1 (Table 1). Unexpectedly, two essential proteins for GABAergic presynapse function, VGAT-2 and synaptotagmin-2, were transiently increased at DIV 10, 5 days after ending the stimulation (Figures 6H,I and Table 1). Also intriguing, Kv3.2, ChAT, and GABA_A_Rα1 were increased at DIV 20, 2 weeks after the stimulation (Table 1). At DIV 10 and DIV 20, total GluN2B was at the control level, but the level of Y1472 phosphorylated GluN2B was lower than control (Figure 6E and Table 1). Another 20 proteins and glutamate receptor phosphorylation sites were not regulated at the time points tested (Table 1). Only GAD-67 and S1480-phosphorylated GluN2B had mild increases, which, however, did not reach robust significance levels. The results suggested that an early postnatal mAChR activation elicits substantial increases—immediate or delayed—of selected proteins. The enhanced expression of GAD-65, VGAT-1, and Syt-2 could argue for an accelerated development of more and/or larger GABAergic synapses.

**Table 1:** Quantitative results of Western blots for proteins altered and not altered by DIV 1-5 CCH.

Expression level altered by DIV 1–5 CCH stimulation	Fold of control mean ± S. E. M. (n) DIV 5	*p*-value	DIV 10	*p*-value	DIV 20	*p*-value
GluN2A	**1.577 ± 0.137 (11)**	**<0.001**	**1.748 ± 0.783 (22)**	**0.005**	1.004 ± 0.086 (28)	n.s.
Syp	**1.562 ± 0.170 (19)**	**0.001**	**1.288 ± 0.089 (34)**	**<0.001**	1.038 ± 0.089 (29)	n.s.
GluN2B	**1.239 ± 0.064 (52)**	**0.001**	1.059 ± 0.066 (40)	n.s.	1.117 ± 0.076 (35)	n.s.
Syt-1	**1.263 ± 0.102 (10)**	**0.01**	1.056 ± 0.179 (12)	n.s.	0.989 ± 0.145 (11)	n.s.
PSD-95	**1.339 ± 0.105 (34)**	**<0.001**	1.036 ± 0.053 (32)	n.s.	0.978 ± 0.037 (18)	n.s.
GluN1	**1.323 ± 0.087 (43)**	**0.003**	1.147 ± 0.086 (38)	n.s	1.102 ± 0.111 (18)	n.s.
VGLUT-1	**1.221 ± 0.086 (17)**	**0.03**	0.938 ± 0.056 (18)	n.s.	0.986 ± 0.069 (15)	n.s.
GAD-65	**1.609 ± 0.230 (23)**	**0.003**	1.109 ± 0.093 (26)	n.s.	1.065 ± 0.079 (21)	n.s.
VGAT-1	1.083 ± 0.132 (4)	n.s.	**1.600 ± 0.173 (22)**	**<0.001**	0.997 ± 0.053 (22)	n.s.
Syt-2	0.953 ± 0.059 (24)	n.s.	**1.262 ± 0.067 (19)**	**<0.001**	1.059 ± 0.049 (30)	n.s.
GABA_A_Rα1	1.045 ± 0.122 (17)	n.s.	1.109 ± 0.047 (30)	n.s.	**1.157 ± 0.059 (25)**	**0.024**
Kv3.2	n.d		1.128 ± 0.132 (10)	n.s.	**1.343 ± 0.140 (20)**	**0.013**
ChAT	n.d		1.064 ± 0.024 (16)	n.s.	**1.332 ± 0.207 (9)**	**0.003**
ph Y1472 GluN2B	1.079 ± 0.091 (17)	n.s.	**0.848 ± 0.032 (31)**	**0.020**	**0.859 ± 0.036 (30)**	**0.004**
**not altered by CCH**						
**GAD-67**	**1.191 ± 0.111 (20)**	n.s.	1.133 ± 0.105 (25)	n.s.	1.009 ± 0.085 (21)	n.s.
**ph S1480 GluN2B**	**1.186 ± 0.165 (16)**	n.s.	0.959 ± 0.063 (8)	n.s.	0.979 ± 0.086 (9)	n.s.
ph Y1246 GluN2A	1.060 ± 0.160 (14)	n.s.	1.021 ± 0.093 (11)	n.s.	1.009 ± 0.070 (20)	n.s.
GluA1	1.039 ± 0.019 (30)	n.s.	0.994 ± 0.033 (35)	n.s.	0.925 ± 0.031 (11)	n.s.
ph S845 GluA1	0.907 ± 0.085 (12)	n.s.	0.919 ± 0.101 (9)	n.s.	0.918 ± 0.166 (5)	n.s.
GluA2	1.040 ± 0.086 (11)	n.s.	1.050 ± 0.066 (21)	n.s.	0.947 ± 0.069 (15)	n.s.
ph S880 GluA2	1.043 ± 0.137 (13)	n.s.	0.906 ± 0.147 (7)	n.s.	0.984 ± 0.093 (6)	n.s.
Synpo	n.d.		1.127 ± 0.087 (9)	n.s.	1.004 ± 0.027 (18)	n.s.
Syn	1.005 ± 0.076 (44)	n.s.	0.982 ± 0.056 (43)	n.s.	0.945 ± 0.105 (15)	n.s.
ph S9 Syn	0.958 ± 0.054 (21)	n.s.	0.887 ± 0.085 (18)	n.s.	0.986 ± 0.085 (16)	n.s.
Kv3.1b	n.d.		0.978 ± 0.048 (18)	n.s.	1.054 ± 0.065 (30)	n.s.
GABA_A_Rα2	1.040 ± 0.063 (15)	n.s.	0.931 ± 0.026 (28)	n.s.	0.945 ± 0.034 (21)	n.s.
GABA_A_Rα3	0.957 ± 0.040 (21)	n.s.	0.937 ± 0.028 (30)	n.s.	1.122 ± 0.063 (20)	n.s.
GABA_A_Rα5	n.d.		1.023 ± 0.056 (12)	n.s.	1.155 ± 0.081 (12)	n.s.
GABA_A_Rβ3	1.068 ± 0.031 (6)	n.s.	1.017 ± 0.052 (19)	n.s.	0.926 ± 0.043 (9)	n.s.
Gephyrin	0.950 ± 0.042 (12)	n.s.	1.017 ± 0.042 (20)	n.s.	1.089 ± 0.69 (15)	n.s.
KCC2 140 kDa	0.912 ± 0.078 (18)	n.s.	0.947 ± 0.048 (25)	n.s.	0.943 ± 0.041 (24)	n.s.
NKCC1	1.176 ± 0.147 (11)	n.s.	1.178 ± 0.074 (21)	n.s.	0.933 ± 0.053 (20)	n.s.
total Src	0.935 ± 0.025 (18)	n.s.	0.911 ± 0.061 (3)	n.s.	0.965 ± 0.027 (24)	n.s.
ph Y418 Src	1.037 ± 0.061 (22)	n.s.	1.026 ± 0.128 (6)	n.s.	0.965 ± 0.071 (30)	n.s.
GAP-43	0.932 ± 0.089 (14)	n.s.	1.084 ± 0.079 (10)	n.s.	0.999 ± 0.029 (9)	n.s.
Thrombospondin	0.940 ± 0.097 (10)	n.s.	1.122 ± 0.147 (12)	n.s.	1.095 ± 0.071 (13)	n.s.
β-actin or β- tubulin	house-keeping					

### Activation of mAChR of subplate/L6b neurons enhances the sensitivity of gray matter neurons to NMDA

The functionality of upregulated NMDA receptor proteins was tested at DIV 5 with a dendritic injury experiment on EGFP-transfected neurons from gray matter layers (Supplementary Figure 4A). Dendritic beading occurs rapidly in neurons overexpressing glutamate receptors upon exposure to the ligand (Hamad et al., 2011; Jack et al., 2019) and in neurons with impaired calcium buffering (Gasterstädt et al., 2020). Mock-stimulated cultures displayed approximately 5% affected neurons. Exposing mock-stimulated OTC to NMDA resulted in a small increase in dendritic beading in polarized cells (Supplementary Figures 4B,C), indicating the functionality of endogenous NMDA receptors. In DIV 1–5 CCH-stimulated OTCs, NMDA exposure increased the fraction of injured cells to approximately 50%, which was blockable by APV (Supplementary Figure 4C). Most affected neurons were polarized, presumptive pyramidal cells, whereas fewer than 20% of multipolar, presumptive non-pyramidal neurons were injured (Supplementary Figure 4D), although we cannot exclude that interneuronal impairment was secondary to pyramidal cell overactivation rather than a direct NMDA effect. Together, the findings demonstrate that the upregulated NMDA receptor protein is functional in the somatodendritic compartment, mainly of immature pyramidal cells.

### Activation of mAChR of subplate/L6b neurons does not influence dendritic growth of gray matter pyramidal cells

The m1 receptor activation has been shown to enhance the development of prenatal hippocampal pyramidal cells at the level of the axon, not minor neurites, and only at very high drug concentration (VanDeMark et al., 2009). However, pyramidal cell dendrites elongate and branch with enhanced glutamatergic activity via AMPAR or TARP overexpression (Hamad et al., 2011; Hamad et al., 2014; Gonda et al., 2020), low doses of kainate or GluK2 overexpression (Jack et al., 2019), optogenetic activation (Gonda et al., 2023a), or chemogenetic activation of Gq signaling (Köhler et al., 2025a). The CCH stimulation evoked synchronous activity in neurons of infragranular layers as early as DIV 3 (Figure 4). During the perinatal period, layer 5 pyramidal cells project to the supragranular compartment, playing an essential role in synchronizing activity within the emerging cortical column (Vargas-Ortiz et al., 2025). Unexpectedly, DIV 1–5 CCH stimulation did not alter the length and branching of apical and basal dendrites of immature pyramidal cells at DIV 5. Similarly, no differences were detected at DIV 10 or DIV 15. Only at DIV 20, a moderate increase in basal dendritic length was seen in L2/3 pyramidal cells (Supplementary Table 2). Overall, the CCH stimulation had little effect on pyramidal cell dendritic architecture. It could suggest that the CCH-evoked activity at perinatal stages was too limited in time compared to the rather harsh genetic manipulations effective in later postnatal periods. Nevertheless, pyramidal cells did show morphological responses at the level of dendritic spines.

### Activation of mAChR of subplate/L6b neurons promotes pyramidal cell spine formation

Spines are highly sensitive to even mild alterations of activity. At DIV 10, total density of spines on apical oblique and basal dendrites was not altered by DIV 1–5 CCH stimulation (L5/6: 19.99 ± 1.21 spines/100 μm in control, 51 cells, versus 20.79 ± 1.04 with CCH, 44 cells; L2/3: 18.13 ± 1.04 in control, 40 cells, versus 16.99 ± 0.94 with CCH, 43 cells). At DIV 10, the spine type ratio of L2/3 pyramidal neurons was shifted to somewhat more spines with large heads (21.6 ± 1.48% in control versus 26.1 ± 2.03% in CCH; MW U-test *p* = 0.135) and significantly less filopodia (10.97 ± 1.04% in control, 50 cells, versus 4.53 ± 0.72% in CCH, 46 cells; MW U-test *p* < 0.001). It suggested that the stronger expression of GluN2A and the disrupted anchoring of GluN2B at synapses have stabilized spines and/or limited spine turnover. At DIV 20, spine density in L2/3 pyramidal neurons had further increased in both groups, consistent with normal maturation, but no longer differed between conditions (29.8 ± 5.7 spines per 100 μm in control, 20 cells, versus 29.1 ± 8.1 in CCH, 37 cells). At DIV 20, the proportion of filopodia further declined (2.01 ± 0.59% in control and 2.14 ± 0.31% in CCH), whereas spines with large heads increased in both groups (36.8 ± 2.01% in control, 28 cells, and 35.01 ± 1.49% in CCH, 72 cells). In accordance, at DIV 20, GluN2A expression was no longer different from control. In summary, early mAChR activation of subplate/L6b neurons transiently accelerated spine stabilization of supragranular neurons at DIV 10, and this difference disappeared by DIV 20 as control neurons caught up with ongoing maturation.

### Activation of mAChR of subplate/L6b neurons promotes the maturation of interneurons

**Figure 7:**
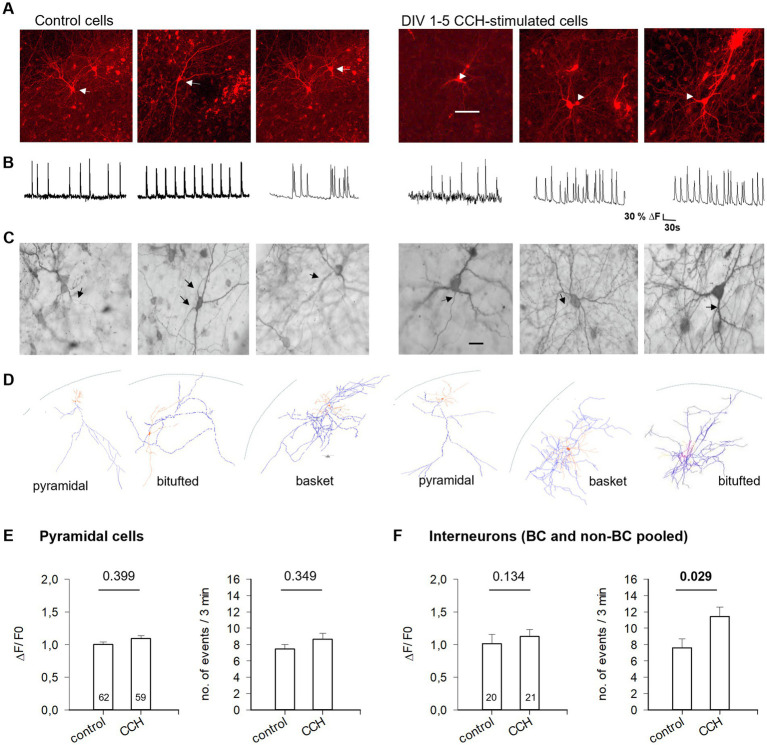
Early CCH stimulation promotes interneuron function. **(A)** Confocal imaging of neurons transfected with mCherry (red), and loaded with OGB at DIV 10–15. Left, three control cells; right, three cells from CCH-stimulated OTCs. **(B)** Spontaneously occurring calcium events displayed by the selected cells. **(C)** The cells were immunostained for classification. **(D)** Partial reconstruction of dendrites (orange) and axons (blue). Cells are not plotted at the same size. The curved lines represent the pial border of the OTCs. **(E)** Amplitude and frequency of calcium events of pyramidal neurons. **(F)** Amplitude and frequency of calcium events of interneurons. Mean ± S. E. M. Given the rather low number of identifiable interneurons, data of basket and non-basket cells were pooled. White arrows in **(A)** point to the selected cell. Black arrows in **(C)** point to the axon; indeed, one cell had two axons.

**Figure 8:**
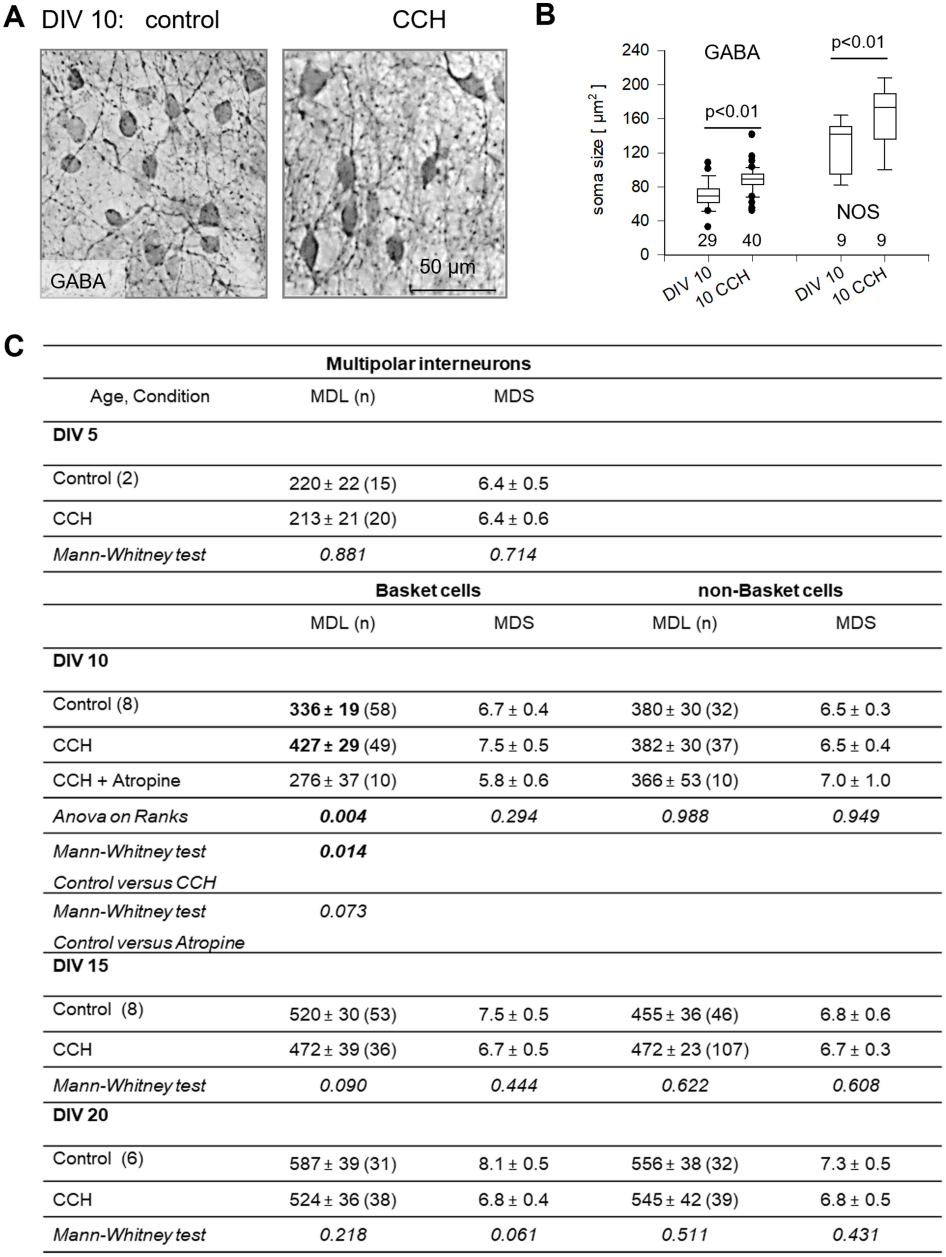
DIV 1–5 mAChR activation promotes somatodendritic differentiation of gray matter interneurons. **(A)** GABA-positive neurons in DIV 10 control and CCH-stimulated OTCs. **(B)** Quantification of soma size of GABA-positive and NOS-reactive neurons. Every dot represents the median size of 50–100 neurons per OTC; the number of OTC is given below the boxes. MWU-tests; *p*-values are given. **(C)** Dendritic parameters of multipolar interneurons in DIV 1–5 CCH-stimulated OTCs. OTCs at DIV 5 were transfected at DIV 2; 2 batches. MWU-tests; *p*-values are given. Significant values bold; MDL, mean dendritic length; MDS, mean dendritic segments, *n* = number of neurons.

The mild effect on pyramidal cells suggested that the CCH-evoked activity is somehow targeted more toward non-pyramidal cells. At DIV 10–15, calcium events of mCherry expressing/OBG-1 loaded or mCherry/GCaMP6 co-expressing neurons (Figures 7A,B) were recorded. Cells were subsequently stained and classified as interneurons and pyramidal cells, and partially reconstructed (Figures 7C,D). DIV 1–5 CCH stimulation did not alter the amplitude or frequency of calcium events of pyramidal cells (Figure 7E). However, DIV 1–5 CCH stimulation led to significantly more calcium events in interneurons (Figure 7F). Moreover, the DIV 1–5 CCH stimulation increased, at DIV 10, the size of GABA-immunoreactive neurons and the size of nitric oxide synthase-positive interneurons (Figures 8A,B). Parvalbumin staining assessed at DIV 15 yielded a similar density of immunopositive somata (123 ± 6.2 cells/mm^2^ in control, *n* = 8,317, 39 OTCs, versus 140 ± 6.4 cells/mm^2^ in CCH, *n* = 9,205 somata, 41 OTCs; MWU-test *p* = 0.061). In the visual cortex *in vivo*, parvalbumin mRNA is upregulated during the second and third week after birth (Patz et al., 2004) and similarly in OTCs (Supplementary Figure 3K), but was not regulated by CCH. Thus, the trend in the MWU-test was rather attributed to a slightly accelerated expression of the parvalbumin protein and an enhanced immunohistochemical detectability.

Dendritic parameters of multipolar, presumptive interneurons were not yet different at DIV 5 (Figure 8C). From DIV 10 onwards, interneurons can be addressed by axonal pattern as basket cells and non-basket cells. Non-basket cell dendrites grew continuously in length at DIV 10, 15, and 20. Neither length nor branching (the number of segments) was altered by CCH and thus, were also not sensitive to atropine. Basket cell dendrites also grew continuously in length from DIV 10 to DIV 20. Intriguingly, at DIV 10, CCH-exposed basket cells displayed longer dendrites, but not more segments, and the effect was sensitive to atropine supplemented from DIV 1–5 (Figure 8C). Also at DIV 10, interneuronal spine density was lower in CCH-treated cells (4.31 ± 0.45 spines per 100 μm in control, 48 cells, versus 3.00 ± 0.35 in CCH, 53 cells, MWU-test *p* = 0.019), suggestive of spines diluting out on elongating dendrites. Assessing presynaptic axonal boutons of DIV 1–5 CCH stimulated basket cells at DIV 15 revealed an increase in the average bouton size per cell (Supplementary Figures 5A–C). Together, early mAChR activation of subplate neurons boosted in particular basket cells, enhancing excitability, dendritic growth, and presynaptic bouton development.

### Activation of mAChR of subplate/L6b neurons promotes axonal growth of gray matter basket cells

**Figure 9:**
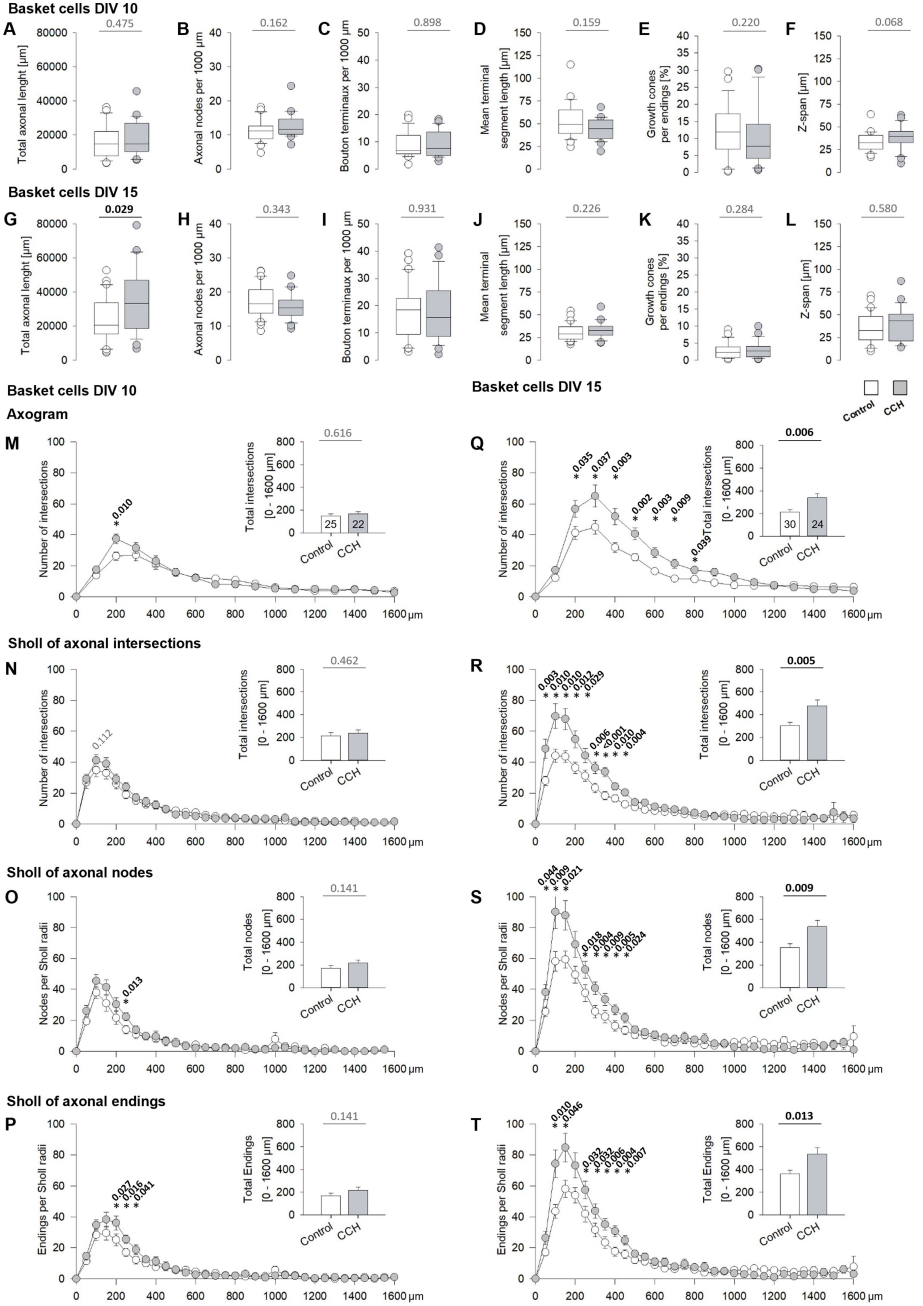
DIV 1–5 mAChR activation promotes differentiation of basket cell axons. **(A–F)** Parameters of axons at DIV 10, and **(G–M)** at DIV 15. **(A,G)** Total axonal length. **(B,H)** Number of axonal branch points (nodes) per 1,000 μm. **(C,I)** Number of Bouton terminaux per 1,000 μm. **(D,J)** Mean length of the terminal segments; the average per cell is plotted. **(E,K)** Proportion of axonal endings with growth cones. **(F,L)** The z-span of the axon plexus within the OTC; the maximal depth value per cell is plotted. **(M–T)** Analyses of axonal branching. Values from the origin of the axon to 1,600 μm distance were considered (arbitrary cut-off; only a few axons reached beyond that distance). **(M–P)** Axons at DIV 10 and **(Q–T)** at DIV 15. **(M,Q)** Linear axogram analysis with 100 μm bins. **(N–P)** Soma-centered Sholl of axonal intersections with 50 μm radius circles at DIV 10, and **(Q–T)** at DIV 15. **(N,R)** Axonal intersections. **(O,S)** Number of nodes and **(P,T)**, the number of axonal endings per 50 μm radius. The small insets report the total intersections, nodes, and endings. *p*-values determined with MWU-tests are given for selected bins and bar graphs; significant *p*-values in bold. The number of axons analyzed in this figure is given in the insets in **(M)** and **(Q)**. For this graph, the CCH condition is toned gray to distinguish the curves.

Basket cell axons were reconstructed in DIV 1–5 CCH-stimulated OTCs at DIV 10 (Figures 9A–F) and at DIV 15 (Figures 9G–L). Total axonal length increased with age, and at DIV 15, axons of basket cells of CCH stimulated OTCs were overall longer (Figures 9A,G). Moreover, the number of nodes per 1,000 μm (Figures 9B,H) increased with age. The number of bouton terminaux, *bona fide* presynapses, was highly variable from cell to cell, albeit the average shifted with age to higher values suggestive of more presynapses (Figures 9C,I). The mean length of the terminal segments forming the perisomatic endings shortened substantially between DIV 10 and DIV 15 (Figures 9D,J), confirming recent data (Gonda et al., 2023b; Köhler et al., 2025b). For comparison, the terminal endings of non-basket cells were much longer (Supplementary Figure 6). Numerous axon collaterals were tipped with growth cones at DIV 10 (Figures 9E,K), and much less at DIV 15. None of these parameters was altered by the CCH stimulation. The z-span of the axon plexus within the OTCs was similar in conditions and at the two ages (Figures 9F,L).

However, the axogram analyses demonstrated clear-cut differences emerging at DIV 10 (Figures 9M–P, left column) and even stronger at DIV 15 (Figures 9Q–T, right column). Linear axogram and Sholl circle measures of intersections, axonal nodes, and terminal endings per 50 μm circle bins revealed that axons from CCH-stimulated OTC were locally more branched than axons from mock-stimulated OTC. This started at DIV 10 within a 200–300 μm distance around the soma and became much more prominent at DIV 15, with branching overshooting that of control axons for >400 μm distance. The increase was not only seen within individual bins, but also in total intersections, nodes, and endings reported as bar graphs in small insets. As expected, the control cell axons displayed an enormous increase in local axonal ramifications between DIV 10–15. Thus, early mAChR activation of cortical subplate neurons selectively accelerated local branching of the axons of basket cells in the cortical gray matter.

### Activation of mAChR of subplate/L6b neurons promotes the growth of somatic axons of basket cells

**Figure 10:**
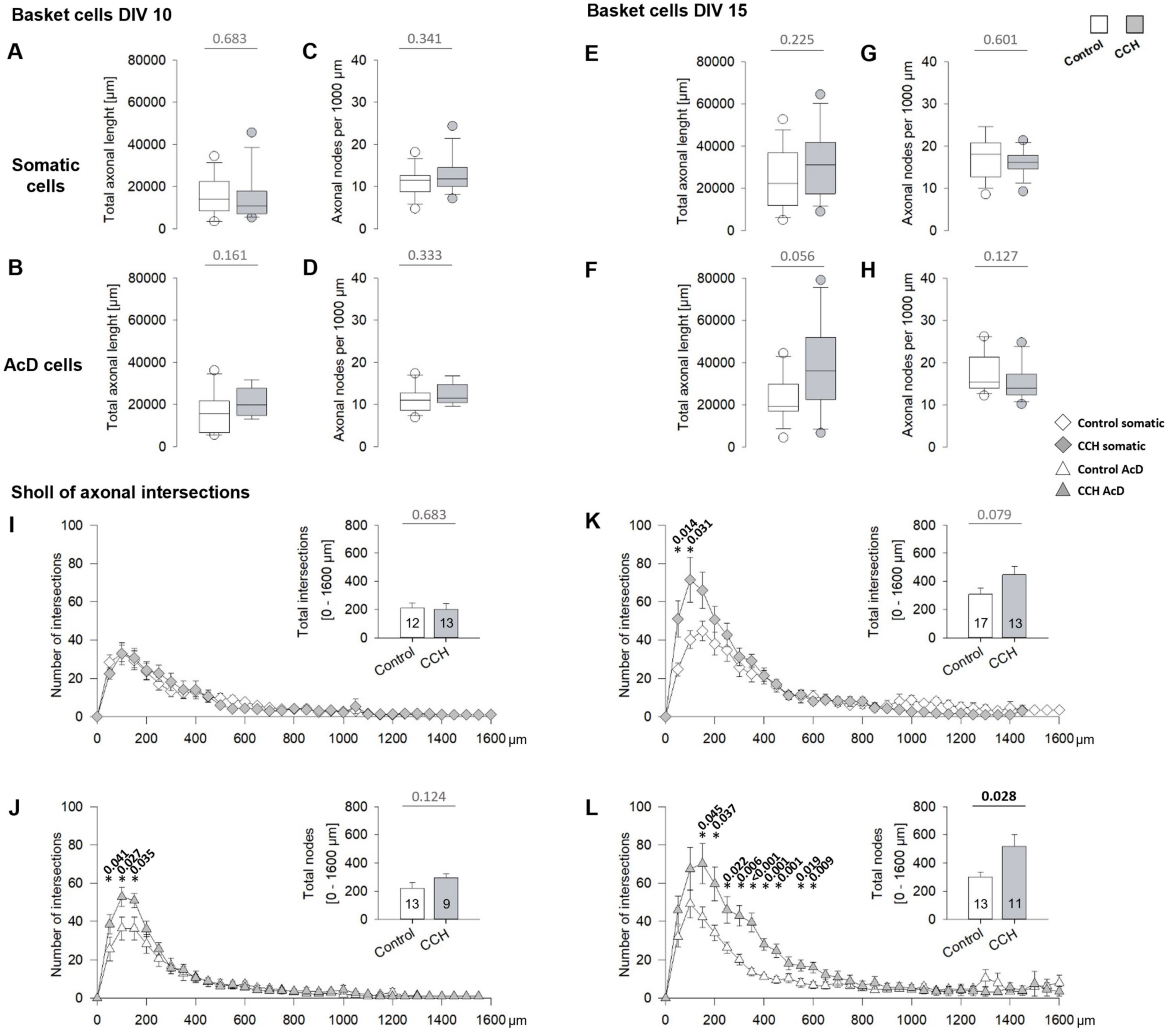
DIV 1–5 mAChR activation promotes differentiation of basket cell axons originating from somata. **(A,C)** Total length and nodes per 1,000 μm of somatic axons at DIV 10. **(B,D)** Total length and nodes per 1,000 μm of axons from an axon-carrying dendrite (AcD) at DIV 10. **(E,G)** Total length and nodes per 1,000 μm of somatic axons at DIV 15. **(F,H)** Total length and nodes per 1,000 μm of AcD axons at DIV 15. **(I)** Sholl of axonal intersections with 50 μm radius circles at DIV 10 for somatic axons, and **(J)** for axons from an axon-carrying dendrite (AcD). **(K)** Sholl of axonal intersections with 50 μm radius circles at DIV 15 for somatic axons, and **(L)** for axons from an AcD. Note the substantial shift in **(L)** to more intersections, which corresponded to the statistical trend to longer AcD axons (in **F**). The small insets in **(I–L)** report the total intersections. *p*-values determined with MWU-tests are given for selected bins and bar graphs; significant *p*-values in bold. The number of axons analyzed in this figure is given in the insets **(I,K)**. The CCH condition is toned gray to distinguish the curves.

These results suggested a network-driven acceleration of the local axonal arborization of basket cells. Such an enhanced arborization can occur in basket cells having axons emerging from a dendrite, the so-called axon-carrying dendrite (AcD). For axons from an AcD, arborization is driven by spontaneous network activity (Gonda et al., 2023b). Mechanistically, glutamatergic activation of GluN2D-containing NMDA receptors promotes basket cell axonal growth (Köhler et al., 2025b). Basket cell axons originating from somata can also develop denser local plexuses, for instance, with optogenetic activation of the cell via channelrhodopsin-mediated depolarization (Gonda et al., 2023b). Therefore, we compared the basket cells with an axon from an AcD and basket cells with a somatic axon selectively. Total length and the number of nodes per 1,000 μm were not different between control cells and cells from CCH-stimulated OTCs at DIV 10 (Figures 10A–D). At DIV 15 (Figures 10E–H), the AcD cells had a clear trend toward longer axons (Figure 10F), indicating that the significant increase of total axonal length at DIV 15 (Figure 9G) is mainly driven by AcD cells. Unequivocal differences emerged with the Sholl analyses (Figures 10I–L). At DIV 10, somatic axon cells had the same degree of axonal arborization in control and CCH-stimulated OTC. However, at DIV 10, axons of AcD cells were more branched up to 200 μm linear distance from the axon origin (Figures 10I,J). This suggested that the increase in the local arborization at DIV 10 is driven by activity, but at this age, it solely promotes basket cell axons emerging from an AcD.

At DIV 15, the axons of AcD cells displayed an enormous increase in the arborization up to 600 μm distance in CCH-stimulated OTC (Figure 10L). Interestingly, at DIV 15, the somatic axon cells also had more local branches within 200 μm distance in the CCH-stimulated OTC (Figure 10K). Together, this indicated that the perinatal mAChR-evoked activation of glutamatergic network activity accelerates the axonal arborization of basket cells, locally of somatic axon cells, and over larger distances of AcD cells.

### Activation of mAChR in subplate/L6b neurons does not promote axonal growth of non-basket cells

Morphometry of non-basket cell axons assessed at DIV 15 in the very same OTC revealed equal length, nodes, bouton terminaux, terminal length, growth cones, as well as the z-span (Supplementary Figures 6A–F). The curves of the axogram and Sholl analyses were completely overlapping (Supplementary Figures 6G–J), indicating that non-basket cells did not respond to the early enhancement of network activity as basket cells did. Non-basket cells with vertical translaminar projections targeting pyramidal cell dendrites frequently have axons from dendrites (Wahle et al., 2022). However, neither the CCH stimulation nor the AcD condition (not shown) led to an enhanced axonal arborization at DIV 10 and at DIV 15.

## Discussion

The study presents evidence that early network activity elicited via a temporally limited perinatal mAChR-mediated activation of subplate neurons triggered a maturation of the GABAergic system and of aspects of pre- and postsynaptic signaling. A first endeavor was the validation of the OTCs since all depended on the presence of the subplate. OTCs assessed for morphometry had a white matter and thus a subplate/L6b by visual inspection and as indicated by the presence of axonal loop cells, inverted, oblique, or horizontal pyramidal neurons, as well as transfected radial glia close to the former ventricular surface. Although the subplate could not be confirmed in OTCs used for molecular analyses, the robust CCH responses make its absence unlikely. If the subplate had been missing, CCH stimulation would have failed to induce activity (Tolner et al., 2012), resulting in an overall different protein expression profile (Lein et al., 1999). The moderate but consistent protein changes we observed align well with previous work in systems not critically dependent on subplate neurons (Engelhardt et al., 2018; Köhler et al., 2025b), suggesting that such an amount of change represents what the network can accomplish at the developmental ages tested.

### Mechanistic considerations

Gq signaling activated by, e.g., m1 receptors couples to the MAP kinase, RhoA, and PI3 kinase to evoke transcriptional and/or translational responses for neuronal proliferation of pyramidal cell progenitors, survival and axonal differentiation of pyramidal cells, and, later postnatal, plasticity with mAChR eliciting protein synthesis in the context of long-lasting depression of excitatory synaptic transmission (Ma et al., 2000; Massey et al., 2001; Abreu-Villaca et al., 2011; Sánchez-Fernández et al., 2014). The possibility that CCH elicits the observed effects via activation of muscarinic receptors directly in the cortical neurons rather than via glutamatergic network activity is unlikely. In primary corticostriatal cultures prepared from P1 rats, mAChR ligand binding sites become detectable earliest at DIV 5 (Eva et al., 1990), which matched our finding of *m1-5* in DIV 5 OTCs. Until DIV 12, m1, m3, and m4 reach intensity levels of the adult cortex (Eva et al., 1990). In the late fetal and newborn cortex *in vivo*, only subplate cells, but not gray matter neurons, display muscarinic binding sites (Schlumpf et al., 1991). The m1 receptor has been shown to elicit MAP kinase phosphorylation in cortical and hippocampal pyramidal cell dendrites (Berkeley et al., 2001). Yet, no growth effect was observed for pyramidal cell dendrites in our study. With regard to inhibitory neurons, it is unknown whether interneurons are responsive to mAChR activation during the first postnatal week. In the mouse cortex at P14, the small subset of so-called multipolar bursting interneurons responds to CCH (Blatow et al., 2003), while the mainly m1-expressing fast-spiking basket neurons (Yi et al., 2014; Disney and Reynolds, 2014) are not responding in P18-22 rat frontal cortex (Kawaguchi, 1997). Moreover, in P15-20 hippocampal basket cells, mAChR reside presynaptically to prevent vesicle depletion during sustained activity (Lawrence et al., 2015). In contrast, non-basket cells with regular- or burst-firing characteristics and mainly expressing m3 partially respond to CCH with depolarization as well as hyperpolarization (Kawaguchi, 1997). Such a heterogeneous response might explain why we could not detect a net growth effect of non-basket cell dendrites and axons at DIV 15. Overall, this suggests that the robust biochemical and structural effects are mediated by glutamatergic network activity.

A further argument against direct actions is that the chemogenetic activation of overexpressed hM3Dq with CNO supplemented from DIV 5–10 causes larger L2/3 pyramidal cell dendritic trees, and an activation from DIV 10–20 reduces dendritic complexity of interneurons (Köhler et al., 2025a). Both effects are the opposite of what we observed with the DIV 1–5 CCH stimulation. Furthermore, at DIV 10, the GABAergic neurons had substantially larger somata in DIV 1–5 CCH-stimulated OTCs. There is no published evidence for mAChR promoting soma growth. Rather, soma growth of GABAergic neurons is a classical consequence of TrkB signaling (Engelhardt et al., 2007) originating from enhanced BDNF production and release driven by glutamatergic depolarization and calcium events in pyramidal cells (Gottmann et al., 2009). Moreover, glutamate receptor signaling and glutamatergic network activity do promote interneuronal differentiation (Hamad et al., 2011; Jack et al., 2019; Köhler et al., 2025b), and ionotropic glutamate receptors are expressed in pyramidal cells and interneurons from fetal stages onwards (Le Magueresse and Monyer, 2013; Hadzic et al., 2017). We do not exclude the possibility that the subplate-driven network activity triggers additional signaling pathways. For instance, the CCH-evoked enhancement of GABAergic neurons reported in the present study may itself contribute to interneuronal differentiation (Chattopadhyaya et al., 2007; Sernagor et al., 2010; Le Magueresse and Monyer, 2013). Furthermore, rat neocortex contains cholinergic neurons, which begin to appear toward the end of the first week (Bruel-Jungerman et al., 2011). Indeed, CHAT was upregulated in CCH-stimulated OTCs, and endogenous acetylcholine may contribute to differentiation via ionotropic nicAChR (Liu et al., 2006).

### Neurochemical maturation

The protein profiling suggested a scenario related to ontogenetic plasticity. The upregulation at DIV 5 of presynaptically enriched GAD-65 argued for a boost of interneuronal maturation. GAD-65 expression depends on activity and TrkB signaling (Patz et al., 2003). Concurrently, the upregulation at DIV 5 of p38/synaptophysin, synaptotagmin-1, VGLUT-1, and PSD-95 suggested an accelerated development of glutamatergic pre- and postsynapses (Knaus et al., 1986; Leclerc et al., 1989). In particular, the amount of PSD-95, which anchors GluN1/GluN2A/B-containing NMDA receptors (Cousins and Stephenson, 2012), correlates with the size and strength of excitatory synapses and the expression level of GluN2A (Ling et al., 2012; Liu et al., 2004). BDNF signaling via TrkB regulates synaptic delivery of PSD-95 (Yoshii et al., 2011). Presynaptically, p38/synaptophysin is enriched in VGLUT-1—positive synaptic vesicles (Grønborg et al., 2010), contributing to biogenesis, release, and endocytosis. The upregulation of GAD-65, VGAT-1, and Syt-2 suggested a boost for GABAergic axon growth and presynapse development. More GAD-65 can produce more GABA in a shorter amount of time, and VGAT transports GABA into synaptic vesicles. Syt-2 is the basket cell-specific calcium sensor essential for the fast-spiking phenotype (Sommeijer and Levelt, 2012). The upregulation of p38/synaptophysin and GluN2A at DIV 10 suggested a further increase of excitatory pre- and postsynapses, which is known to occur during the second postnatal week. It concurred with a downregulation of Y1472 GluN2B phosphorylation at DIV 10 and DIV 20, which suggests a de-anchoring of GluN2B subunits at synaptic sites and a shift toward internalization or to extrasynaptic sites. The latter could well explain the lethal sensitivity of DIV 5 neurons to NMDA. Matching the interpretation is the mild increase of S1480-phosphorylated GluN2B. This occurs indirectly via calcium-regulated CaMKII, which brings CK-2 to phosphorylate GluN2B, and disrupting these interactions reduces synapse numbers and increases synaptic GluN2B content (Sanz-Clemente et al., 2013). Together, this could tip the balance toward GluN2A-containing receptors, which become more prominent during the precritical and critical period in vivo via a decline of synaptic GluN2B rather than a dramatic increase of GluN2A, also because Y1246 GluN2A phosphorylation was unchanged, as was activation of c-Src, which phosphorylates GluN2A.

Also fitting to the concept was the upregulation at DIV 20 of Kv3.2, ChAT, and the GABA_A_Rα1 subunit. Kv3.2 is a delayed rectifier potassium channel in interneurons essential for firing non-adapting action potentials at high frequencies. Kv3.2 is expressed in infragranular FS neurons and supragranular dendrite-targeting non-FS non-basket cells and is regulated by activity (Rudy and McBain, 2001; Grabert and Wahle, 2009). ChAT is expressed in bipolar interneurons, which inhibit selectively other interneurons, mainly of the non-basket types, to regulate disinhibition of pyramidal cell dendrites. Inhibitory postsynapses enriched for the GABA_A_Rα1 subunit increase during the precritical period in rodent visual cortex, are a prerequisite for ocular dominance plasticity in visual cortex and thalamus (Fagiolini et al., 2004; Sommeijer et al., 2017), and the upregulation has been previously shown in cat cortex to depend on activity triggered by subplate neurons (Kanold and Shatz, 2006).

### Role of BDNF and activity-dependent transcription

The initial CCH-evoked surge in BDNF and immediate early gene expression was short-lived, suggesting that transient trophic signaling alone cannot explain the delayed, highly selective upregulation of selected proteins at later stages. Accelerated maturation of inhibition occurs with an early postnatal excess of TrkB ligands, which in the visual cortex leads to an earlier closure of the critical period (Huang et al., 1999; Berardi et al., 2003; Hensch, 2005). On the other hand, it is difficult to believe that the delayed upregulation of proteins related to inhibition, or the downregulation of Y1472 GluN2B, has been evoked by the short transient surge of, e.g., BDNF 2 weeks before. Rather, also given that the proteins have variable half-lifetimes and turnover rates, e.g., in the range of hours for PSD proteins (Gray et al., 2006) or synaptic NMDAR (Soltesz et al., 1994), the possibility exists that the perinatal mAChR activation and subsequently enhanced glutamatergic input to gray matter neurons have led to an activity-dependent transcriptional or translational reprogramming of the neurons.

### Structural maturation

The increase in markers for excitatory synapses corresponded to the reduction of dendritic filopodia at DIV 10. Apparently, turnover has slowed, and filopodia have become stabilized presumably via the enhanced expression of PSD-95 and GluN2A-containing receptors (De Roo et al., 2008) in the absence of changes in the basal and apical dendritic arbors of pyramidal cells. The latter was puzzling because earlier studies have demonstrated a role for glutamatergic activity in pyramidal cell dendritic growth. We speculate that the CCH-evoked network activity might have been no longer strong enough during the period of pyramidal cell growth around DIV 10–15 to remodel dendritic patterns. In fact, calcium event amplitude and frequency were similar in control and CCH-exposed pyramidal cells at DIV 10–15. In particular, the amplitude of glutamatergic events is a crucial driver of dendritic remodeling (Hamad et al., 2011; Hamad et al., 2014). Also, the CCH-evoked enhancement of GABAergic neurons might have counteracted. Further, previous work has shown that an optogenetic stimulation @0.05 Hz from DIV 5–10 is not strong enough to remodel pyramidal cell dendritic trees, whereas a stimulation @0.5 Hz does promote dendritic growth (Gonda et al., 2023a).

A major finding was the increase in local axonal arborization of basket cells. Their axons have been shown to be highly dynamic during development and into adulthood (Micheva et al., 2021). The increase emerged at DIV 10 concurrent with the enhanced expression of VGAT-1 and Syt-2 and became highly significant at DIV 15, the major period of basket cell axon differentiation in the visual cortex. For comparison, the somatosensory cortex is developmentally more advanced, as described above with the higher peak of *Bdnf*, a known growth factor for interneurons. Accordingly, in mouse S1, dendrite-targeting (non-basket) interneuronal synapses have formed as early as P5, while axosomatic (basket) synapses gradually emerge between P5-P9 (Gour et al., 2021). In our study of the visual cortex at DIV 10 and DIV 15, the CCH exposure evoked more complex arborizations locally for axons emerging from somata, and over larger distances around the parent somata for axons emerging from an AcD. We presume the AcD configuration works in synergy with the enhanced network activity. The results are fully in line with our earlier reports. Optogenetic stimulation of basket cells promotes the arborization of axons with somatic origin to a similar degree to what the AcD condition can elicit in the absence of optogenetic stimulation, solely with spontaneously occurring network activity (Gonda et al., 2023b). The results confirm the view that glutamatergic network activity is a prerequisite for the axonal growth of basket cells’ axons emerging from the soma or from an AcD. Further, it confirmed the activity dependence of basket cell axon growth (Chattopadhyaya et al., 2007). Our findings demonstrate that activating network activity via mAChR stimulation can naturally elicit this effect, possibly via involvement of GluK2/Neto2 (Jack et al., 2019) and/or GluN2D-containing NMDA receptors (Köhler et al., 2025b).

## Conclusion

In summary, it was intriguing to see that a low-dose mAChR activation perinatally for a few days had such a substantial effect on transcription, translation, and/or protein stability with both acute changes as well as changes that gradually unfold over the following weeks. Moreover, it highly selectively led to local arborization of basket cell axons. Thus, the cholinergic input to the perinatal cortical subplate plays an important role in the neurochemical and structural development of cortical interneurons.

## Data Availability

The raw data supporting the conclusions of this article will be made available by the authors, without undue reservation.
