# Supplementary material for: Perinatal mAChR-mediated activation of cortical subplate neurons elicits network activity driving basket cell differentiation
Source: Front Cell Neurosci. 2026 May 14;20:1799121. doi: 10.3389/fncel.2026.1799121 (PMC13215935; doi:10.3389/fncel.2026.1799121)
Supplement: Supplementary file 1 [file Data_Sheet_1.pdf]

Supplementary material

“Perinatal mAChR-mediated activation of cortical subplate neurons elicits network activity driving basket cell differentiation”

Petra Wahle, Michelle Kaczmarski, Christian Riedel, Mohammad I. K. Hamad, Olga Arne, Erwan Dupont, Alexander Jack, Lisa-Marie Rennau, Andrea Räk, Heiko J. Luhmann, Ina Köhler, Silke Patz

Supplementary Table 1. Plasmids, antibodies, reagents

Material	Species	Source	cat. no. and/or RRID	Method, Dilution	Antigen (~kDa)
**Plasmids**					
pEGFP-N1 in pcDNA3.0	-	Clontech, Heidelberg, Germany	632370	-	-
pAAV-mDlx-GCaMP6f-Fishell-2	-	Addgene	83899	-	-
pAAV-mDlx-GFP-Fishell-1	-	Addgene; Dimidsch-stein et al. 2016	83900	-	-
pCMV-mCherry-N1	-	Clontech, Heidelberg, Germany	632523	-	-
pGP-CMV-GCaMP6m	-	Addgene	40754	-	-
**Primary antibodies**					
β-actin	mouse	Merck	A1978<br>RRID: AB_476692	WB<br>1:2000	40
βIII-tubulin	mouse	Merck	T8660<br>RRID: AB_477590	WB,<br>1:10000	55
PSD 95	rabbit	Synaptic Systems	124011<br>RRID: AB_10804286	WB,<br>1:1000	95
GluN1	mouse	NeuroMab	75-272<br>RRID: AB_11000180	WB<br>1:1000	130
GluN2B	rabbit	Merck	06-600<br>RRID: AB_310193	WB,<br>1:1000	180
GluN2B	mouse	NeuroMab	75-097<br>RRID: AB_10673405	WB<br>1:2000	180
phospho Y1472 GluN2B	rabbit	Calbiochem/Millipore	454583<br>RRID: AB_262150	1:1000	180
GluN2A	mouse	NeuroMab	75-288<br>RRID: AB_2315842	WB<br>1:1000	180
phospho Y1246 GluN2A	rabbit	Cell Signaling	4206<br>RRID: AB_2112292	1:1000	180
phospho S1480 GluN2B	rabbit	Rockland/BioMol	612-401-D93<br>RRID: AB_11181871	1:1000	180
GluA2	mouse	NeuroMab	75-002<br>RRID: AB_2232661	WB<br>1:1000	130
phospho S880 GluA2	rabbit	Rockland/BioMol	612-401-D64<br>RRID: AB_11182371	WB<br>1:1000	130
GluA1	mouse	NeuroMab	75-327<br>RRID: AB_2315840	WB<br>1:1000	130
phospho S845 GluA2	rabbit	Rockland/BioMol	612-401-C83<br>RRID: AB_11181875	WB<br>1:1000	130
GAD 65/67	mouse	Enzo Life Sciences	ADI-MSA-225E<br>RRID: AB_2039129	WB,<br>1:1500	65/67
Synaptotagmin-1	mouse	Synaptic Systems	105011<br>RRID: AB_887832	WB<br>1:1000	67
Synaptotagmin-2	rabbit	Synaptic Systems	105225,<br>RRID: AB_2744654	WB,<br>1:1000	67

Synaptotagmin-2	guinea pig	Synaptic Systems	105225<br>RRID: AB_2744654	WB,<br>1:2000	67
VGAT (cytosolic domain)	rabbit	Synaptic Systems	131002<br>RRID: AB_887871	WB<br>1:1000	57
VGLUT-1	rabbit	Synaptic Systems	135302<br>RRID: AB_887877	WB<br>1:3000	65
Kv3.1b	Rrabbit	Synaptic Systems	242003<br>RRID: AB_11043175	WB<br>1:1000	110
KV3.2	rabbit	Alomone	APC.011<br>RRID: AB_20401686	WB<br>1:1000	70
ChAT	goat	Chemicon/Sigma	AB144P<br>RRID: AB_2079751	WB<br>1:1000	70
Synaptophysin	mouse	Synaptic Systems	101011<br>RRID: AB_887824	WB<br>1:1000	38
Synaptopodin	rabbit	Synaptic Systems	163002<br>RRID: AB_887825	WB,<br>1:1000	100
Synapsin	mouse	Synaptic Systems	106011,<br>RRID: AB_2619772	WB,<br>1:2000	78
phospho S9 synapsin	rabbit	Rockland/BioMol	612-401-C93<br>RRID: AB_11182681	WB<br>1:1000	78
GABA(A)R α1	mouse	NeuroMab	75-136<br>RRID: AB_ 2877288	WB,<br>1:700	52
GABA(A)R α2	guinea pig	Synaptic Systems	224104<br>RRID: AB_10639393	WB<br>1:1000	52
GABA(A)R α3	rabbit	Synaptic Systems	224302<br>RRID: AB_2619930	WB<br>1:1000	60
GABA(A)R α5	rabbit	Synaptic Systems	224502<br>RRID: AB_2619943	WB<br>1:1000	60
GABA(A)R β3	mouse	NeuroMab	75-149<br>RRID: AB_2109585	WB<br>1:1000	55
Gephyrin	mouse	Synaptic Systems	147111<br>RRID: AB_887719	WB<br>1:1000	93
c-Src	rabbit	Santa Cruz	SC-18<br>RRID: AB_631324	WB<br>1:1000	60
phospho 418 c-Src	rabbit	Invitrogen	446606	WB<br>1:1000	60
ERK 1/2	mouse	Santa Cruz	Sc-135900<br>RRID: AB_2141283	WB<br>1:1000	44/42
phospho-ERK 1/2	Mouse	Merck	M8159<br>RRID: AB_477245	WB<br>1:1000	44/42
phospho-ERK 1/2	rabbit	New England Biolabs	9101S	WB,<br>1:1000	44/42
GFAP	rabbit	DAKO A/S	Z0334<br>RRID: AB_10013382	WB<br>1:2000	50
GAP-43	rabbit	abcam	ab75810<br>RRID: AB_1310252	WB<br>1:1000	50
activated/total Ras	rat	Gift of Rolf Heumann	clone Y13-259	IP/WB	21
NeuN	mouse	Sigma	MAB377<br>RRID: AB_2298772	IHC/IF, WB<br>1:1000	44-48
c-Fos	rabbit	Santa Cruz	SC-52<br>RRID: AB_2106783	IHC/IF<br>1:400	-
EGFP	mouse	Merck	clone GSN24	IHC	-

			RRID: AB_563117	1:1000	
mCherry	mouse	TakaraBio	632543<br>RRID: AB_2307319	IHC<br>1:1000	-
GABA	mouse	ICN Biomedicals Inc. or Chemicon	69-328<br>MAB316	IHC/IF<br>1:2000	-
GABA	rabbit	Sigma	A2052	IHC/IF<br>1:2000	-
NPY	rabbit	Sigma	N9528	IHC<br>1:1000	-
Parvalbumin	rabbit	Synaptic Systems	195002<br>RRID: AB_2156474	IHC<br>1:500	-
**Secondary antibodies**					
anti-rabbit biotinylated	goat	Dako A/S	E0432<br>RRID: AB_2313609	IHC<br>1:750	-
anti-mouse biotinylated	goat	Dako A/S	E0433<br>RRID: AB_2687905	IHC<br>1:750	-
Goat serum	goat	Vector	S1000<br>RRID: AB_2336615	IHC/IF<br>5-10%	-
anti-mouse Alexa 488	goat	Thermo-Fisher Scientific	A-11001<br>RRID: AB_2534069	IHC/IF<br>1:1000	-
anti-rabbit Alexa 594	goat	Thermo-Fisher Scientific	A-11012<br>RRID: AB_2534079	IHC/IF<br>1:1000	-
anti-mouse-AP	rabbit	Dako A/S	D0314	WB<br>1:5000	-
anti-rabbit-AP	goat	Dako A/S	D0487<br>RRID: AB_2617144	WB<br>1:2000	-
**Reagents, drugs**					
ABC reagent	-	Vector Laboratories	PK-7100<br>RRID: AB_2336827	as recom-mended	-
Bovine serum albumine	-	Serva	11920.03	5%	-
NADPH-Tetranatrium	-	Roth	AE14.1	as recom-mended	-
BCIP	-	Applichem	A1117	as recom-mended	-
NBT	-	Applichem	A1243	as recom-mended	-
CCH	-	Sigma	C2409	as indicated	-
Atropine	-	Sigma	A0132	as indicated	-
NMDA	-	Sigma	M3262	as indicated	-
APV	-	Sigma	A5282	as indicated	-

Supplementary Reference: Dimidschstein J, Chen Q, Tremblay R, Rogers SL, Saldi GA, Guo L, Xu Q, Liu R, Lu C, Chu J, Avery MC, Rashid MS, Baek M, Jacob AL, Smith GB, Wilson DE, Kosche G, Kruglikov I, Rusielewicz T, Kotak VC, Mowery TM, Anderson SA, Callaway EM, Dasen JS, Fitzpatrick D, Fossati V, Long MA, Noggle S, Reynolds JH, Sanes DH, Rudy B, Feng G, Fishell G. 2016. A viral strategy for targeting and manipulating interneurons across vertebrate species. Nat Neurosci. 19:1743-1749. 10.1038/nn.4430.

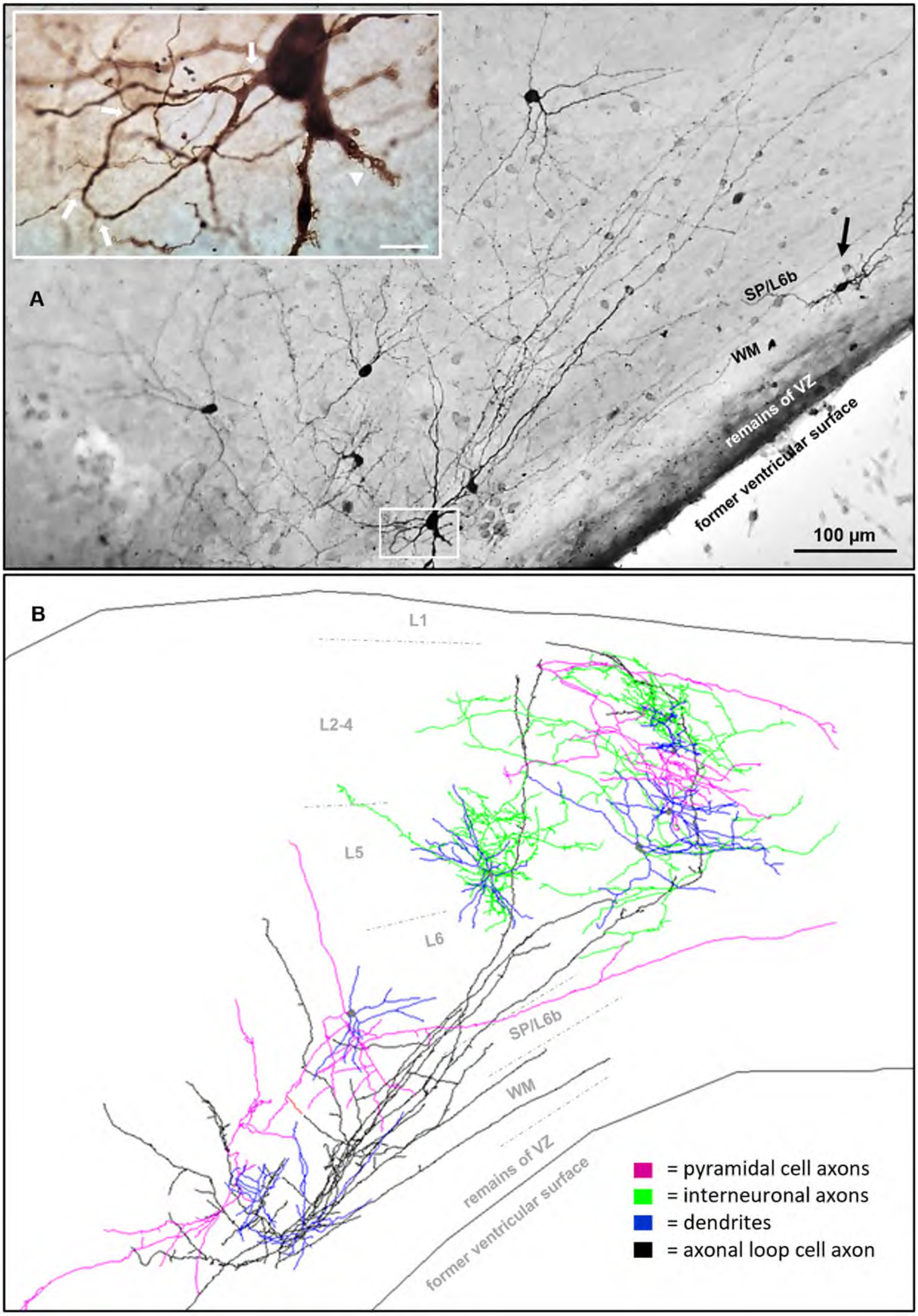
A
SP/L6b
WM
remains of VZ
former ventricular surface
100 µm
B
L1
L2-4
L5
L6
SP/L6b
WM
remains of VZ
former ventricular surface
= pyramidal cell axons
= interneuronal axons
= dendrites
= axonal loop cell axon

Supplementary Fig. 1. Subplate axonal loop cell in OTC. A. EGFP transfected neurons in infragranular layers and subplate close to the remains of the ventricular zone (VZ) of the OTC. The axonal loop cell (white box) is shown at higher magnification in the inset. Arrows point to axon and collaterals, arrowhead points to symptoms of dendritic degeneration. A horizontally oriented pyramidal cell of the white matter (WM) is indicated by the black arrow. B. Neurolucida reconstruction of the axonal loop cell and its long-range projections (axon in black). The collaterals in the gray matter were in close apposition to neurites of pyramidal cells and local axon interneurons (color-coded) of the gray matter L2-6.

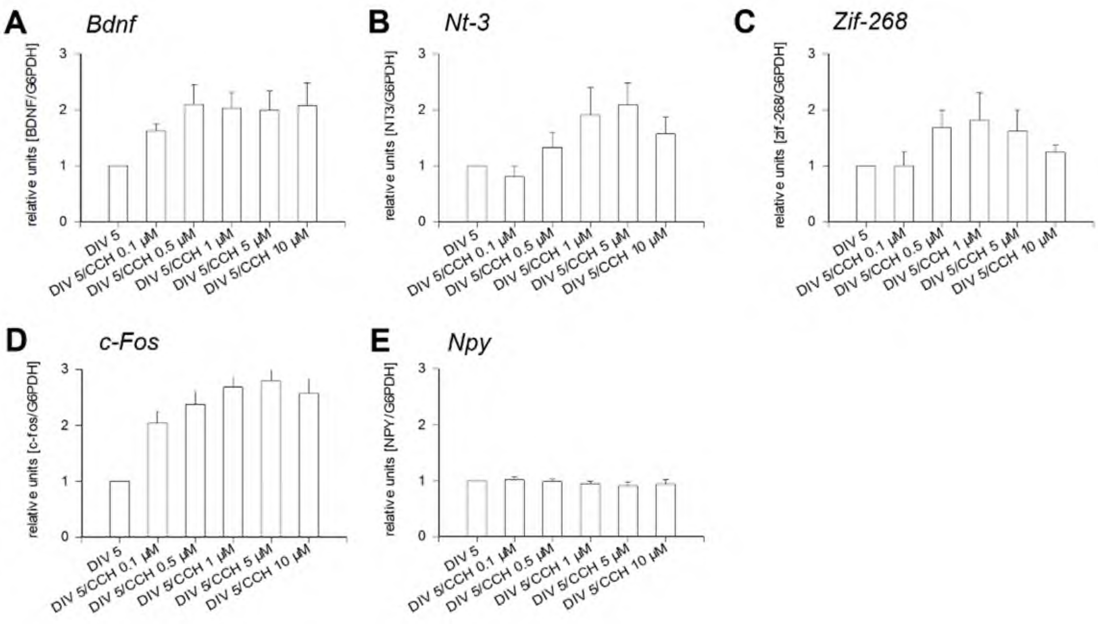

Supplementary Fig. 2. Dose-dependency. Expression of mRNA in DIV 5 OTCs stimulated with 0.1-10 µM CCH for 1 h. (A) *Bdnf*; (B) *Nt*-3; (C) *c-Fos*; (D) *Zif-268*, (E) *Npy*. Note that plateau levels of expression were already evoked by 0.5-1 µM CCH. Three lysates each of 5 OTCs per time point, 3 reactions per lysate, values normalized to G6PDH and averaged. Mean±S.E.M., DIV 5 untreated control was set to 1.

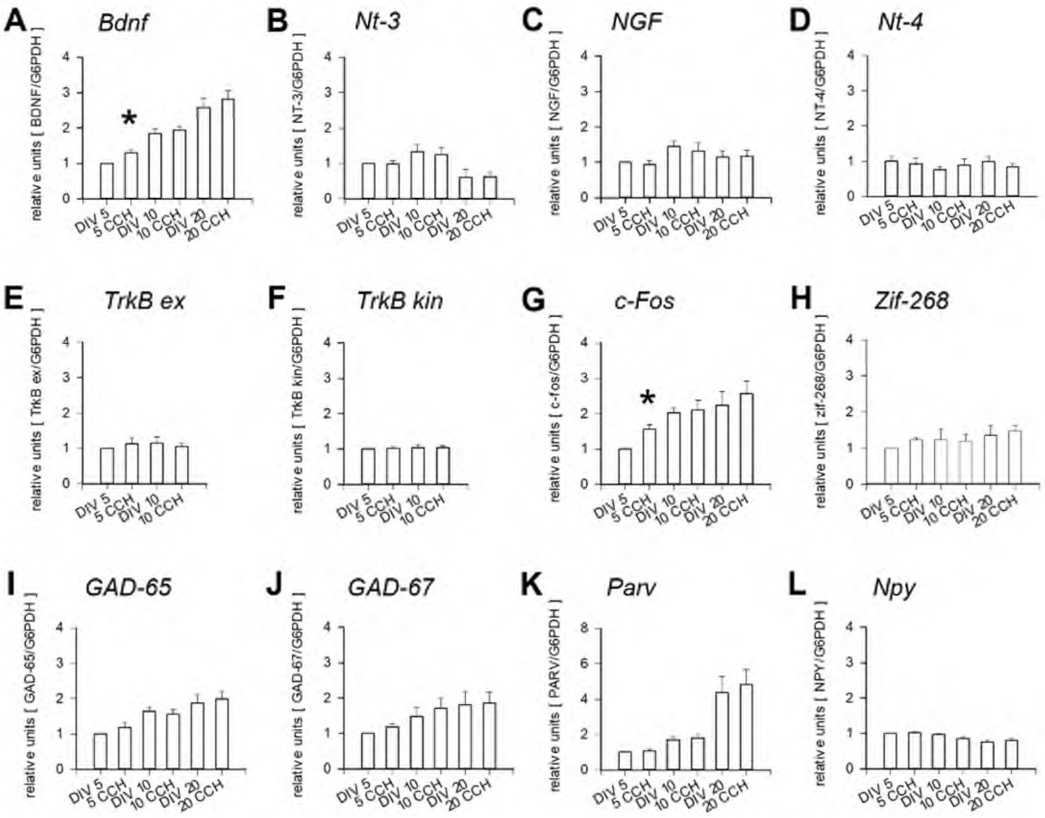

Supplementary Fig. 3. DIV 1-5 mAChR activation does not evoke long-term changes of mRNA expression. (A) *Bdnf*; (B) Nt-3; (C) *Ngf*; (D) *Nt-4*; (E) *TrkB* total/ex, extracellular domain; (F) *TrkB* full length/kin, kinase domain; (G) c-Fos; (H) Zif-268; (I) Gad-65; (J) Gad-67; (K) parvalbumin (*Parv*); (L) *Npy* mRNA. Three lysates each of 5 OTCs per time point, 3 reactions for every product per lysate, values normalized to G6PDH and averaged. Mean±S.E.M., DIV 5 mock-stimulated control was set to 1; t-test for 1 µM versus mock-stimulated control with * $p<0.05$.

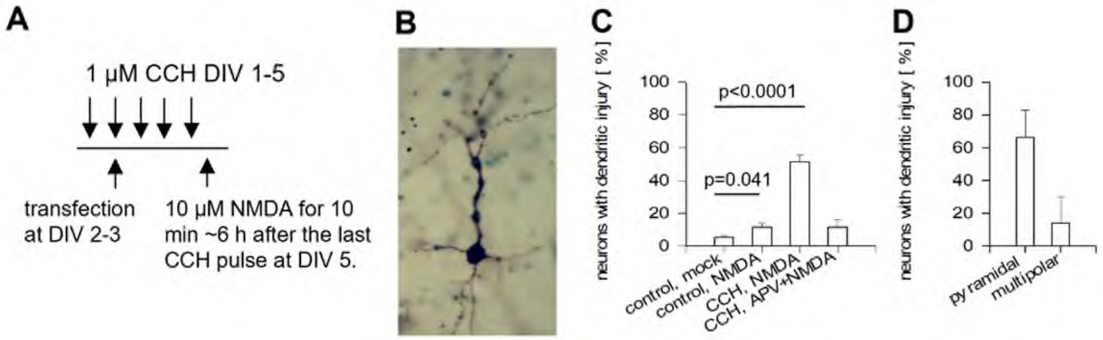

Supplementary Fig. 4. NMDA receptor-evoked dendritic injury. (A) Experimental design. (B) Immature pyramidal neuron from infragranular layers displaying symptoms of excitotoxicity like swellings in apical and in basal dendrites. (C) Proportion of neurons with dendritic injury from total EGFP-stained neurons in L2-6. Within 10 min exposure to 10 µM NMDA around 50% of the CCH-exposed EGFP transfectants displayed dendritic beading mostly along the apical dendrite because basal dendrites are still very immature. The effect was prevented by a 10 min preincubation with 10 µM APV. (D) In the CCH+NMDA group, affected neurons were mainly polarized, presumably pyramidal cells. Of the multipolar cells, presumptive interneurons, less than 20% displayed dendritic injury. MWU-tests; p values are given. Mock-stimulated control: 28 OTCs, 926 neurons; control+NMDA: 33 OTCs, 1342 neurons; CCH+NMDA: 30 OTCs, 1305 neurons; CCH+APV+NMDA: 20 OTCs, 726 neurons; OTCs from 4 independent preparations.

Supplementary Table 2. DIV 1-5 mAChR activation does not promote dendritic differentiation of pyramidal neurons. Dendritic parameters of pyramidal neurons in DIV 1-5 CCH-stimulated OTCs at DIV 5, 10, 15, 20. Neurons were analyzed in L2/3 and L5/6. Mann-Whitney U-tests; p values are given. MDL, mean dendritic length; MDS mean dendritic segments, n = the number of neurons. MWU-tests; p values are given; significant p values bold; n = the number of neurons.

	Pyramidal cells in L2/3		Pyramidal cells in layers L5/6	
Age, Condition (Number of batches)	ADL (n) Segments	BDL Segments	ADL (n) Segments	BDL Segments
**DIV 5**				
Control (2)	619 ± 49 (27)	113 ± 13	581 ± 44 (26)	111 ± 12
	22.6 ± 1.8	4.2 ± 0.4	15.5 ± 1.8	3.7 ± 0.5
CCH	569 ± 44 (21)	100 ± 16	565 ± 47 (15)	121 ± 14
	20.7 ± 1.5	3.2 ± 0.5	16.3 ± 1.5	3.7 ± 0.4
Mann-Whitney test	*0.461*	*0.257*	*0.989*	*0.425*
	0.699	*0.061*	*0.447*	*0.383*
**DIV 10**				
Control (8)	931 ± 34 (61)	187 ± 14	1077 ± 45 (66)	192 ± 14
	24.0 ± 1.0	6.1 ± 0.4	25.5 ± 1.2	5.4 ± 0.4
CCH	942 ± 35 (55)	174 ± 14	961 ± 41 (58)	203 ± 13
	24.9 ± 1.1	5.5 ± 0.3	24.6 ± 1.2	6.0 ± 0.4
CCH + Atropine	895 ± 86 (16)	147 ± 17	993 ± 76 (22)	193 ± 23
	21.1 ± 2.3	5.3 ± 0.5	20.6 ± 2.1	6.0 ± 0.9
Anova on Ranks	*0.711*	*0.282*	*0.223*	*0.536*
	0.189	*0.621*	*0.106*	*0.243*
**DIV 15**				
Control (4)	1533 ±137 (25)	305 ± 31	1102 ± 97 (20)	316 ± 28
	29.5 ± 2.8	6.3 ± 0.7	22.1 ± 2.7	6.6 ± 0.7
CCH	1521 ± 110 (39)	365 ± 34	1197 ± 94 (22)	343 ± 43
	30.4 ± 2.8	7.7 ± 0.7	23.1 ± 2.0	6.0 ± 0.7
Mann-Whitney test	*0.962*	*0.231*	*0.473*	*0.980*
	0.804	*0.253*	*0.527*	*0.597*
**DIV 20**				
Control (6)	1654 ± 69 (51)	**338 ± 27**	1570 ± 89 (42)	343 ± 21
	33.5 ± 1.9	8.1 ± 0.6	28.2 ± 2.2	7.4 ± 0.5
CCH	1758 ± 66 (92)	**392 ± 18**	1578 ± 54 (59)	394 ± 23
	33.3 ± 1.5	9.0 ± 0.4	27.7 ± 1.5	7.9 ± 0.5
Mann-Whitney test	*0.398*	***0.017***	*0.682*	*0.149*
	0.988	*0.101*	*0.922*	*0.644*

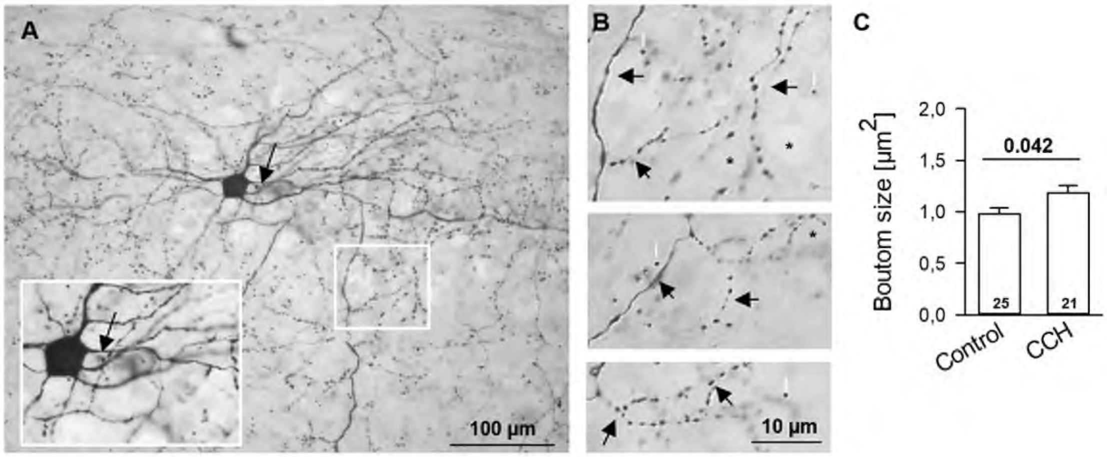

Supplementary Fig. 5. DIV 1-5 mAChR activation promotes morphological differentiation of basket cell axonal boutons. (A) Basket cell within its axonal domain with presynaptic boutons. Inset shows soma and axon origin at higher magnification. (B) DIC-optics showing the main axon and, typically, the delicate collaterals (small arrows) studded with irregular sized boutons. The boxed area in (A) is shown top. Asterisks mark unstained somata enwrapped by terminal elements. The fine vertical lines mark gold particles (1 µm) used for biolistics; a perfect internal scale. (C) EGFP bouton size in μm^2 at DIV 15 (average per cell is plotted; 2283 boutons in control, 1565 in CCH); the number of neurons is indicated in the bars.

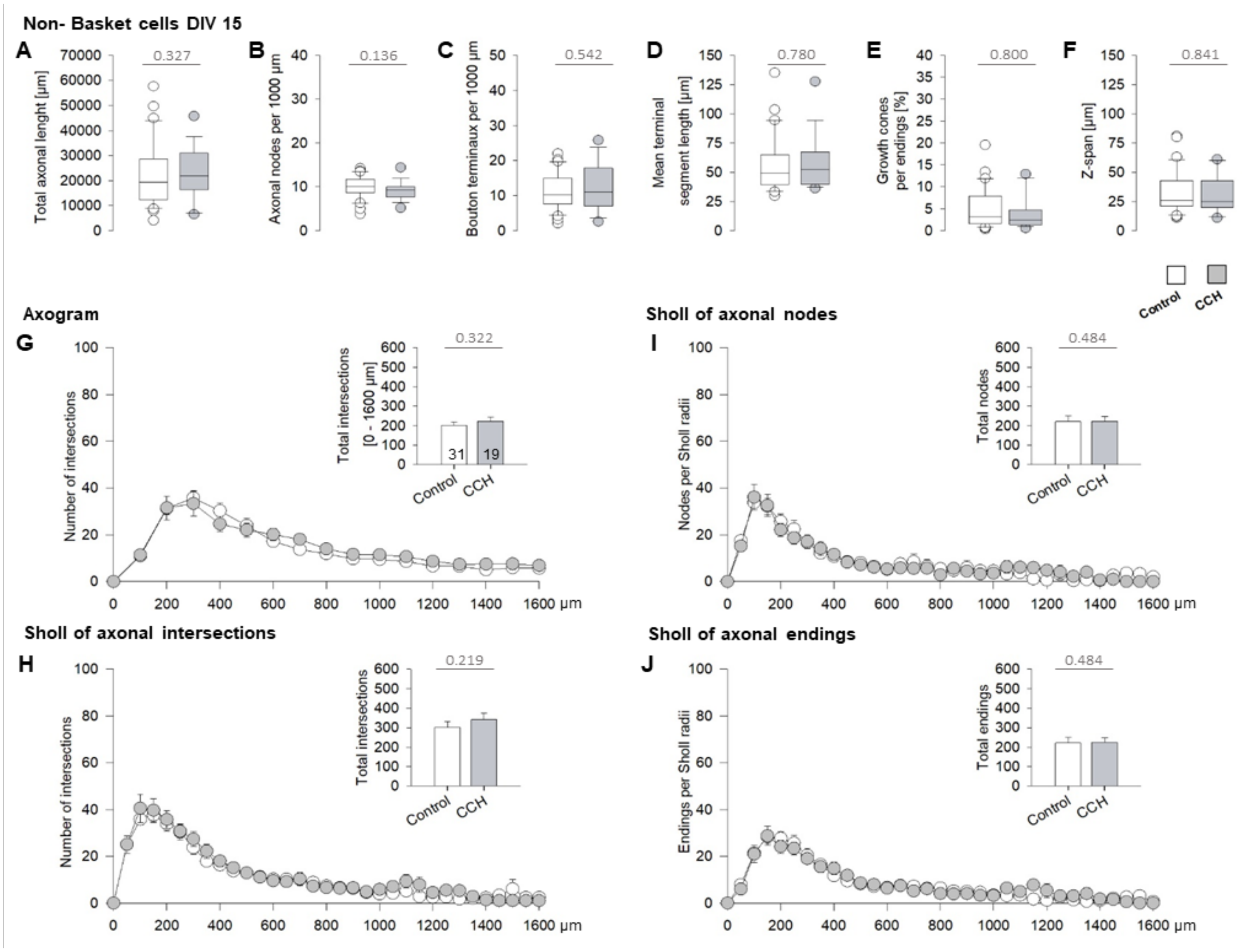

Supplementary Fig. 6. DIV 1-5 mAChR activation does not promote axonal differentiation of non-basket cells. (A-F) Parameters of axons at DIV 15. (A) Total axonal length. (B) Number of axonal branch points (nodes) per 1000 µm. (C) Number of Bouton terminaux per 1000 µm. (D) Mean length of the terminal segments, the average per cell is plotted. (E) Proportion of axonal endings tipped with growth cones. (F) The z-span of the axon plexus; the maximal depth value per cell is plotted. (G-K) Analyses of axonal branching at DIV 15. Values from the origin of the axon to 1600 µm distance were considered (arbitrary cut-off, only a few axons reached beyond that distance). (G) Linear axogram analysis. (H-J) Soma-centered Sholl of axonal intersections with 50 µm radius circles. (H) Axonal intersections. (I) The number of nodes and (J) the number of axonal endings per 50 µm radius. The small insets report the total intersections, nodes, and endings. The number of axons is given in the inset in (G). For this graph, the CCH condition is toned gray to distinguish the curves.
